# TME-responsive nanocomposite hydrogel with targeted capacity for enhanced synergistic chemoimmunotherapy of MYC-amplified osteosarcoma

**DOI:** 10.1016/j.bioactmat.2025.01.006

**Published:** 2025-01-14

**Authors:** Yichao Ma, Peng Lai, Zhou Sha, Bing Li, Jiangpeng Wu, Xiaojun Zhou, Chuanglong He, Xiaojun Ma

**Affiliations:** a Department of Orthopedics, Shanghai General Hospital, Shanghai Jiao Tong University School of Medicine, Shanghai, 200080, China; b College of Biological Science and Medical Engineering, Donghua University, Shanghai, 201620, China; c Shanghai YangZhi Rehabilitation Hospital (Shanghai Sunshine Rehabilitation Center), School of Medicine, Tongji University, Shanghai, 200092, China

**Keywords:** MYC-amplified osteosarcoma, Nanocomposite hydrogel, Tumor microenvironment, Targeted delivery, Combination therapy

## Abstract

The oncogene MYC is one of the most commonly activated oncogenic proteins in human tumors, with nearly one-fourth of osteosarcoma showing MYC amplification and exhibiting the worst clinical outcomes. The clinical efficacy of single radiotherapy, chemotherapy, and immunotherapy for such osteosarcoma is poor, and the dysregulation of MYC amplification and immune-suppressive tumor microenvironment (TME) may be potential causes of anti-tumor failure. To address the above issues, we developed an injectable TME-responsive nanocomposite hydrogel to simultaneously deliver an effective MYC inhibitor (NHWD-870) and IL11Rα-targeted liposomes containing cisplatin-loaded MnO_2_ (Cis/Mn@Lipo-IL11). After *in situ* administration, NHWD-870 effectively degrades MYC and downregulates CCL2 and IL13 cytokines to trigger M1 type activation of macrophages. Meanwhile, targeted delivery of Cis/Mn@Lipo-IL11 reacts with excess intratumoral GSH to generate Mn^2+^ and thus inducing excess active oxygen species (ROS) production through Fenton-like reaction, along with cisplatin, thereby inducing immunogenic cell death (ICD) to promote dendritic cell maturation. Through synergistic regulation of MYC and ICD levels, the immune microenvironment was reshaped to enhance immune infiltration. In the osteosarcoma-bearing model, the nanocomposite hydrogel significantly enhanced tumor T cell infiltration, induced effective anti-tumor immunity and attenuated lung metastasis. Therefore, our results reveal a powerful strategy for targeted combination therapy of MYC-amplified osteosarcoma.

## Highlights

• An injectable TME-responsive nanocomposite hydrogel was developed for targeted delivery of MYC inhibitor and cisplatin.
• TEM-responsive nanocomposite hydrogel was able to control MYC and ICD levels for the treatment of MYC-amplified osteosarcoma.
• An effective therapeutic strategy was presented for targeted therapy of MYC-amplified osteosarcoma growth and metastasis.

## Graphical abstract

We have developed an injectable TME-responsive nanocomposite hydrogel loaded with an effective MYC inhibitor NHWD-870 and IL11Ra-targeted liposomes containing cisplatin-loaded MnO_2_, establishing a targeted chemotherapy-chemodynamic therapy-immunotherapy triple-combination therapy to restrain cancer growth. By regulating the levels of MYC and ICD, the proportion of M1 macrophages and mature dendritic cells were enhanced and thereby strengthening anti-tumor immunity to inhibit MYC-amplified osteosarcoma growth and metastasis.

**Figure:**
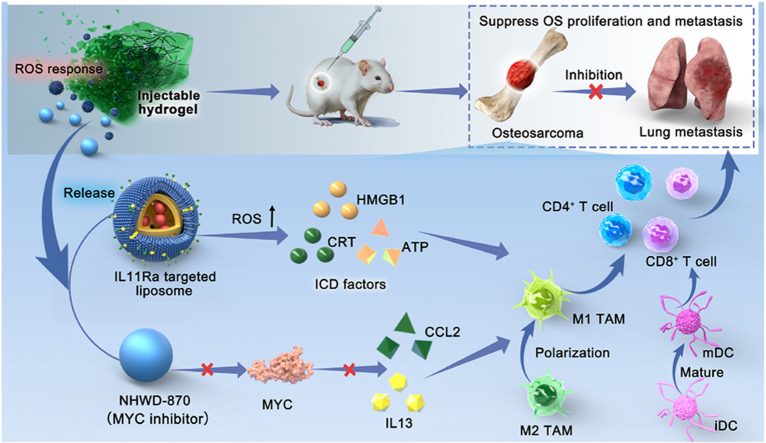

## 1 Introduction

Osteosarcoma (OS) is one of the most common primary malignant tumors during childhood and poses a significant threat to the health of young individuals [1]. In the past 40 years, there has been no substantial improvement in the 5-year survival rate of OS. The main reasons for this include the high heterogeneity, invasiveness, and metastatic nature of OS and the limited effectiveness of conventional radiotherapy and chemotherapy interventions for OS [2,3]. Multiple pieces of evidence indicate that the oncogene MYC can promote the occurrence, progression, and metastasis of various malignant tumors, including glioblastoma, triple-negative breast cancer, and OS. Additionally, MYC can affect the tumor immune microenvironment through multiple pathways [[4], [5], [6]]. Previous studies have reported that MYC amplification occurs in close to one-fourth of OS patients. MYC-amplified osteosarcoma has been identified as the most malignant subtype, with a 5-year survival rate of less than 40 %. These tumors are also associated with low immune infiltration, frequent chemotherapy resistance, and lung metastasis [[7], [8], [9]]. Silencing MYC using a small molecule drug NHWD-870 is a potential targeted therapy approach that can make OS sensitive to platinum-based chemotherapy and inhibit the growth and metastasis of OS [10,11]. Indeed, some studies have suggested that abnormally high levels of MYC may promote resistance to immunotherapy, particularly by inhibiting the extrinsic immune response against tumor cells [[12], [13], [14]]. It is encouraging that targeting MYC inhibition has synergized with PD-1 blockade therapy in inhibiting tumor metastasis and recurrence in MYC-positive cells [[15], [16], [17]].

In the tumor microenvironment (TME), dysfunctional tolerogenic dendritic cells (DCs) and tumor-associated macrophages (TAMs) - especially tumorigenic M2-like TAMs - have been shown to promote immune evasion and are associated with poor clinical outcomes in various cancers [[18], [19], [20]]. Early evidence suggests that MYC regulates the transformation of M1-like macrophages into M2-like macrophages by promoting the expression of cytokines such as C-C motif chemokine ligand 2 (CCL2) and Interleukin-13 (IL13) [21]. Furthermore, the concept of immunogenic cell death (ICD) is an important mechanism driving tumor immunogenicity in current clinical treatments. ICD enhances anti-tumor immune responses by releasing damage-associated molecular patterns (DAMPs) that stimulate dendritic cell maturation and cytotoxic T cell activity. This process attracts various immune cells to the tumor microenvironment, transforming "cold" tumors into "hot" tumors [[22], [23], [24]].

In addition, the development of nanotechnology has brought new vitality to the efficacy of cancer treatment. The small size of nanoparticles allows them to penetrate biological barriers and deliver drugs to tumor sites, achieving higher treatment efficiency. Nanoparticles can also be functionally targeted, prioritizing the delivery of drugs to tumor sites and minimizing side effects [[25], [26], [27]]. Interleukin 11 receptor alpha (IL11Rα) is highly expressed in OS, especially in high-risk or advanced-stage patients, and its high expression is associated with poor prognosis in OS [28]. In a preliminary study, it was found that by modifying the specific peptide of IL11Rα onto the oxidoreductive responsive polymer vesicles loaded with doxorubicin (IL11-PDox), the specificity and therapeutic efficacy against OS were successfully enhanced [29]. Nanocomposite hydrogels possess a three-dimensional structure that can achieve various anti-tumor properties by incorporating multifunctional nanomaterials. One recognized advantage of hydrogel-based medical interventions is their ability to control in-situ degradation, which can be utilized for precise and long-term release of individual components from nanocomposite hydrogels [[30], [31], [32], [33]]. Similar techniques have been attempted in the field of OS [34,35]. TME-responsive nanomedicines have been demonstrated to safely and effectively enhance specific and localized immune responses in tumor tissues, thereby improving the safety and efficacy of drugs while reducing immune-related side effects [36,37]. High levels of reactive oxygen species (ROS) are an essential characteristic of tumor tissues, which is also significantly expressed in OS. Based on this feature, various related anti-tumor strategies have been developed [[38], [39], [40]], including (1) further increasing ROS levels in tumor cells to reduce drug resistance and (2) constructing ROS-responsive nanomedicines to enhance drug accumulation in tumors. However, the effect of ROS-responsive nanomedicines in promoting tumor regression is not ideal due to insufficient local ROS supply in tumors. Therefore, exogenous or endogenous stimuli must be introduced to enhance ROS supply. Manganese dioxide (MnO_2_) has TME-responsive degradation properties, effectively releasing Mn^2+^ under the action of excessive glutathione (GSH) in the microenvironment and generating hydroxyl radicals (·OH) [[41], [42], [43]]. Therefore, introducing Mn^2+^ in nanotherapeutic systems can increase ROS levels in the tumor microenvironment, thereby regulating drug release in ROS-responsive nanosystems and reducing drug resistance in cancer cells.

**Fig. 1:**
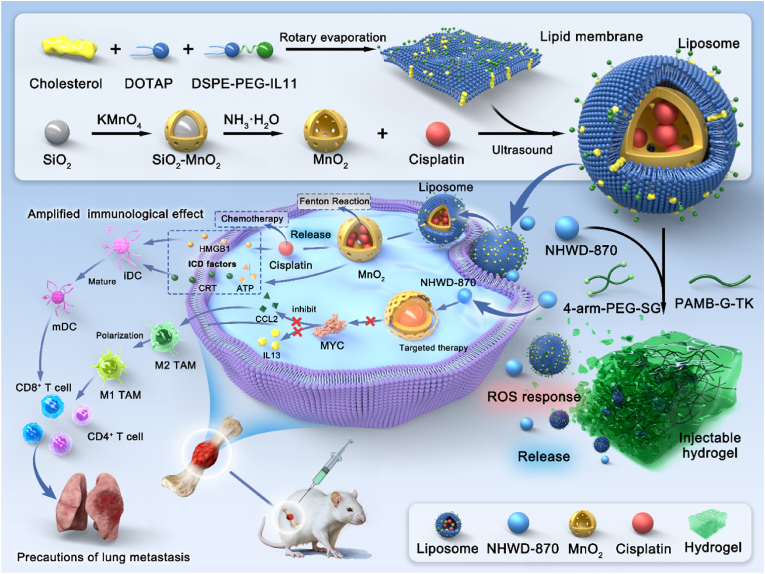
Schematic diagram of the preparation and application of Cis/Mn/NH@PGP. An injectable TME-responsive nanocomposite hydrogel loaded with an effective MYC inhibitor (NHWD-870) and IL11Rα-targeted liposomes containing cisplatin-loaded MnO_2_ (Cis/Mn@Lipo-IL11) was developed, establishing a targeted chemotherapy-chemodynamic therapy-immunotherapy triple-combination therapy to treat MYC-amplified osteosarcoma. By regulating the levels of MYC and ICD, the proportion of M1 macrophages and mature dendritic cells were enhanced and thereby strengthening anti-tumor immunity to inhibit MYC-amplified osteosarcoma growth and metastasis.

In this study, we aimed to develop a multifunctional nanocomposite that targeted multiple vulnerabilities of MYC-amplified OS for achieving enhanced therapeutic efficacy. Here, an injectable ROS-responsive nanocomposite hydrogel (Cis/Mn/NH@PGP) was designed through the incorporation of nanosystem into injectable hydrogel. This nanocomposite hydrogel is dynamic and responds to the tumor microenvironment of MYC-amplified OS, which consists of free NHWD-870 (NH) and IL11Rα-targeted liposomes containing cisplatin-loaded MnO_2_ (Cis/Mn@Lipo-IL11), providing powerful targeted therapy and immune modulation against OS progression and metastasis (Fig. 1). After intratumoral injection, the hydrogel can gradually degrade under the high ROS conditions in the TME and release the targeted MYC therapy drug (NHWD-870) and Cis/Mn@Lipo-IL11. The small molecule drug NHWD-870 is released to target MYC therapy and inhibit the production of downstream cytokines CCL2 and IL13, promoting the polarization of tumorigenic M2 macrophages to anti-tumor M1 macrophages. The liposomes encapsulated with cisplatin-loaded mesoporous MnO_2_ target tumor cells through surface-modified IL11Rα. Subsequently, the GSH in the TME promotes the formation of Mn^2+^, leading to a Fenton-like reaction that generates ROS for chemodynamic therapy. Particularly, released cisplatin and Mn^2+^ induce ICD to promote the maturation of DCs. These mature DCs and M1 macrophages further activate T cells, improving overall immune infiltration and suppressing tumor growth and metastasis. The results show that the TME-responsive nanocomposite hydrogel possesses enhanced anti-tumor efficacy in MYC-amplified OS, which supports further development of rational combination therapy for MYC-amplified tumor treatment.

## 2 Materials and methods

### 2.1 Preparation of hydrogel

Poly-3-amino-4-methoxybenzoic acid gelatin (PAMB-G) was synthesized using oxidative polymerization method by mixing 3-amino-4-methoxybenzoic acid (AMB) and gelatin at a ratio of 1:10 (w/w). The specific procedure is as follows: 1 g of gelatin and 100 mg of AMB were dissolved in 10 mL of deionized water and thoroughly mixed at 40 °C. Then, 136.5 mg of ammonium persulfate (APS) was added as the catalyst for oxidative polymerization. After stirring for 24 h, the reaction solution was dialyzed in deionized water for 3 days and freeze-dried to obtain PAMB-G. To synthesize poly(3-amino-4-methoxybenzoic acid)-gelatin-thioketal (PAMB-G-TK), thioketal (TK) linkages were conjugated with PAMB-G to achieve a ROS-responsive conjugate. Specifically, 300 mg of PAMB-G and 100 mg of 2-[2-(2-aminoethylsulfanyl)propan-2-ylsulfanyl]ethanamine (TK-NH_2_) were precisely dissolved in DMSO. Then, 0.25 g of 1-(3-Dimethylaminopropyl)-3-ethylcarbodiimide (EDC) was added, and the mixture was stirred at 37 °C for 24 h. Finally, after extensive dialysis against PBS (pH 7.4) with a molecular weight cutoff (MWCO) of 3.5 kDa and freeze-drying, PAMB-G-TK was obtained. For the preparation of 3%PAMB-G-TK/1.5%PEG-SG (PGP) hydrogel, 30 mg of PAMB-G-TK was dissolved in 800 μL PBS to obtain a clear solution A, and 15 mg of 4-arm-PEG-SG were dissolved in 200 μL PBS to obtain a clear solution B. Solution B was then added to solution A, and the resulting mixture was quickly gelled and maintained at 37 °C for a short period. Next, 5 mg of the previously synthesized Cis/Mn@Lipo-IL11 (quantified by cisplatin) and 5 mg of NHWD-870 were added to 250 mg of PGP (3 %/1.5 %). Then, 1 mL of deionized water was added to the mixture, and it was subjected to ultrasonic dispersion to produce the complete Cis/Mn/NH@PGP.

### 2.2 Determination of MYC and IL-11Rα expression on cell lines

For determination of MYC and IL-11Rα expression in different tumor cell lines, 143B, HOS, SJSA and U2OS were cultured. Subsequently, cell lysates were prepared using radioimmunoprecipitation assay (RIPA) lysis buffer (#P0013, Beyotime, China) to extract proteins, and their concentrations were determined using the Bicinchoninic Acid (BCA) Protein Assay Kit (#23227, Thermo Fisher Scientific, USA). Equal amounts of protein were separated by gel electrophoresis, followed by protein-blots analysis to evaluate the expression of MYC proteins. Similarly, the cell lines 143B, L929, HUVECs and NIH3T3 were collected after incubation. The proteins extracts were prepared using RIPA lysis buffer and their concentrations were measured using the BCA Protein Assay Kit. Afterward, the expression of IL-11Ra proteins were evaluated by protein-blots analysis.

### 2.3 Cellular uptake of Cis/Mn@Lipo-IL11

Assessment of cellular uptake behavior of Cis/Mn@Lipo-IL11 was conducted through flow cytometry and confocal laser scanning microscopy (CLSM). 143B and L929 cells were cultured separately in 12-well plates or CLSM-specific culture dishes. After overnight incubation, the supernatant was replaced with fresh culture medium containing Lipo-IL11 and Lipo with Cyanine5.5 (Cy5.5) labelling at predetermined time points. Cells in the 12-well plate were washed three times with PBS and then resuspended in PBS for flow cytometry analysis. For CLSM identification, cells in the culture dishes were fixed with 4 % paraformaldehyde for 15 min, followed by staining with DAPI and FITC-labeled phalloidin (5 μg/mL, 15 min) to visualize the cell nucleus and cytoskeleton.

### 2.4 In vitro therapeutic efficacy evaluation

143B cells were incubated with PBS, Lipo-IL11, Mn@Lipo-IL11, Cis/Mn@Lipo-IL11, Cis/Mn@Lipo-IL11+NH, and Cis/Mn/NH@PGP (100 μg/mL) for 24 h. After an additional 24 h of incubation, cell viability was assessed using the Cell Counting Kit-8 (CCK-8 assay). Relative cell viability was calculated based on the control group treated with PBS. Additionally, cell viability was assessed using Calcein-AM/PI staining under inverted fluorescence microscopy (Carl Zeiss, Germany).

### 2.5 RAW264.7 polarization inducted by Cis/Mn/NH@PGP

A 24-well transwell system with a 0.4 μm porous membrane was utilized. 143B cells were cultured in the upper chamber and treated with different materials for 24 h. Collecting culture medium, levels of the cytokines CCL2 and IL-13 in the culture medium after treatment with different materials were first measured using ELISA kits. The RAW264.7 cell line was subsequently collected from the lower chamber and analyzed after co-staining with CD206-FITC, CD86-PE, and IgG isotype controls using flow cytometry.

### 2.6 Detection of ICD markers

K7M2 cells were treated with PBS, Lipo-IL11, Mn@Lipo-IL11, Cis/Mn@Lipo-IL11, Cis/Mn@Lipo-IL11+NH, and Cis/Mn/NH@PGP for 24 h. Collecting culture medium, levels of the cytokines HMGB1 in the culture medium after treatment with different materials were first measured using ELISA kits. Then, the cells were incubated with Alexa Fluor 488-conjugated anti-CRT antibody (10 μg/mL, 30 min). Subsequently, CRT expression release were observed using CLSM. ATP secretion induced by therapeutic drugs was detected using the ATP Assay Kit (#S0026, Beyotime, China). K7M2 cells were seeded in 6-well plates at a density of 2 × 10^5^ cells/well and treated with PBS, Lipo-IL11, Mn@Lipo-IL11, Cis/Mn@Lipo-IL11, Cis/Mn@Lipo-IL11+NH, and Cis/Mn/NH@PGP for 24 h. ATP concentration in the culture medium was determined according to the manufacturer's instructions.

### 2.7 Induction of ICD-mediated DCs maturation

A 24-well transwell system with a 0.4 μm porous membrane was utilized. K7M2 cells were cultured in the upper chamber and DCs were seeded in the lower chamber. After the K7M2 cells were treated with different materials for 24 h, DCs were collected and stained with PE-anti-CD80 and APC-anti-CD86, and then analyzed by flow cytometry.

### 2.8 Evaluation of STING pathway activation *in vitro*

DCs were seeded in the lower chamber of 24-well transwell system and collected after 24 h. Subsequently, cell lysates were prepared using RIPA lysis buffer to extract proteins, and their concentrations were determined using the BCA Protein Assay Kit. Equal amounts of protein were separated by gel electrophoresis, followed by protein-blots analysis to evaluate the expression of STING, TBK1 and IRF3 proteins.

### 2.9 Establishment of *in situ* 143B and subcutaneous K7M2 tumor models

In situ 143B OS tumor models were established using Balb/c nude mice (5–6 weeks). 143B cells were harvested and suspended in PBS at 4 °C. All the mice were anesthetized with isoflurane. Before the right hind limb of mice was incised, iodophor was wiped to remove bacteria. Then, the mice's knees were bent more than 90° and a 29-gauge needle was rotated through the proximal tibial cortex. Once the tibial bone cortex was penetrated, 5 × 10^5^ 143B cells were injected into the tibia under anesthesia. The subcutaneous K7M2 OS tumor models were established using Balb/c mice (5–6 weeks). K7M2 cells were harvested and suspended in PBS at 4 °C. All the mice were anesthetized with isoflurane. Then, 5 × 10^5^ K7M2 cells were injected into the subcutaneous groin of the mice with a 29-gauge needle. When the primary tumor volume reached approximately 50 mm^3^, the tumor-bearing mouse models were used for *in vivo* experiments.

### 2.10 Evaluation of *in vivo* tumor accumulation and biodistribution

In the 143B OS mouse model, Lipo-IL11 and Lipo with Cy5.5 labelling (200 μg/mL) were intravenously injected (n = 3). At 0, 12, 24, 36, and 48 h after nanoparticles injection, *in vivo* real-time fluorescence imaging was performed to assess the accumulation of nanoparticles in tumor. At 48 h post-injection, mice were euthanized, and fluorescence analysis was conducted on kidneys, liver, lungs, spleen, heart, and tumors. Regions of interest (ROIs) were used to quantify the fluorescence intensity in tumors at different time points and in various organs.

### 2.11 In vivo therapeutic efficacy evaluation

The 143B OS and K7M2 mouse model was randomly divided into 6 groups and treated with PBS, PGP, Mn@PGP, Cis/Mn/NH@PGP, Mn/NH@PGP, Cis/Mn/NH@PGP (25 mg/kg) by intratumoral injection (n = 5). From day 0 to day 24, tumor size and body weight of the mice were recorded every 3 days. After treatment, the mice were euthanized, and tumors were extracted from the mice for H&E, Ki67, MYC, IL11Rα and TUNEL staining to investigate their anti-tumor effects.

In the 143B OS mouse model, all mice were euthanized on day 24. The excised tumors were subjected to micro-CT scanning (Quantum FX, PerkinElmer, USA) for assessing bone destruction.

After 24 days of treatment in K7M2 OS mouse model, the mice were euthanized, and primary tumors were extracted to measure intratumoral ATP levels. To assess the expression levels of CRT and HMGB1, primary tumors were extracted from the mice after 24 days of treatment, frozen sectioned, and stained with corresponding antibodies. Fluorescence images were obtained using CLSM. Additionally, after 24 days of treatment, the mice were euthanized, and primary tumors were extracted to measure intratumoral IL13 and CCL2 levels.

### 2.12 Evaluation of *in vivo* immune response in mice

Tumor-draining lymph nodes and spleens of mice were harvested and digested with collagenase to acquire a single cell suspension (n = 5). The cells were then stained with Cy5.5-*anti*-CD3, FITC-anti-CD4, and PE-anti-CD8 and the flow cytometry analysis was performed to detect the content of CD4^+^ and CD8^+^ T cells. For the analysis of macrophages, the single cell suspension was stained with PE-anti-F4/80, FITC-anti-CD86 and APC-anti-CD206 antibodies. For the analysis of DCs, the single cell suspension was stained with FITC-anti-CD86 and APC-anti-CD11c antibodies. For the analysis of tumor-infiltrated regulatory T cells (Tregs), the cell suspension was stained with FITC-anti-CD4, PE-anti-CD25 and APC-anti-Foxp3 antibodies. To analyze memory T cells, the single cell suspensions harvested from spleens were respectively stained with PE-anti-CD8, FITC-anti-CD44 and APC-anti-CD62L antibodies to differentiate central memory T cells (T_CM_, CD8^+^CD44^+^CD62L^+^) and effector memory T cells (T_EM_, CD8^+^CD44^+^CD62L^−^). In addition, the cytokines in sera including TNF-α, IL-6, IFN-γ were measured using ELISA kits. Immunofluorescence staining in tumor tissues for CD4^+^ and CD8^+^ T cells was conducted through staining with anti-CD4 and anti-CD8.

### 2.13 Evaluation of anti-metastatic effects *in vivo*

The K7M2 OS mouse model was randomly divided into 6 groups and treated with PBS, PGP, Mn@PGP, Cis/Mn/NH@PGP, Mn/NH@PGP, Cis/Mn/NH@PGP (25 mg/kg) (n = 5). After 24 days, the mice were euthanized, and lung tissues were collected for H&E staining to observe tumor metastasis. To quantify lung metastases, the number of tumor nodules in each lung was recorded.

### 2.14 Statistical analysis

All quantitative data in this study are presented as the mean ± standard deviation (SD) of at least three independent experiments (n ≥ 3). Statistical analysis was performed using Student's t-test (two-tailed) and one-way analysis of variance (ANOVA). ∗P < 0.05, ∗∗P < 0.01, ∗∗∗P < 0.001. All statistical analyses were conducted using GraphPad Prism 9.0 (GraphPad Software, USA) or SPSS 22.0 (IBM, USA).

## 3 Results and discussion

### 3.1 Preparation and characterization of Cis/Mn@Lipo-IL11

**Fig. 2:**
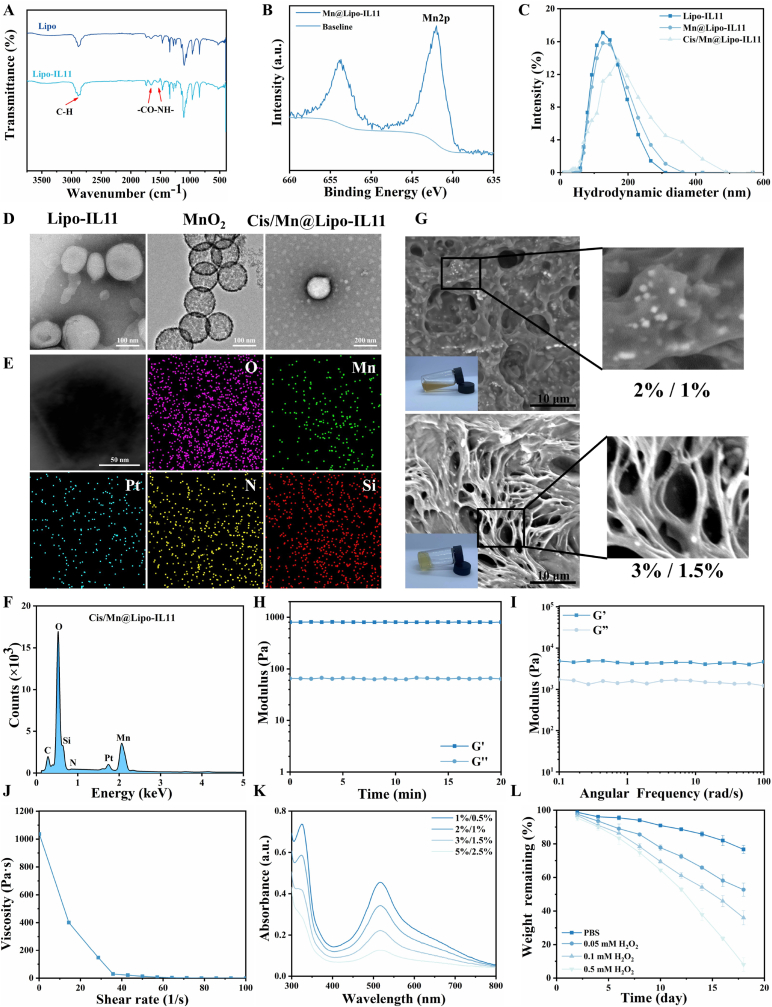
Preparation and characterization of Cis/Mn/NH@PGP. (A) FTIR spectra of Lipo and Lipo-IL11. The peaks corresponding to the C-H, C=O and N-H vibrations were enhanced in Lipo-IL11 as compared to Lipo. (B) XPS spectrum of Mn@Lipo-IL11. (C) Particle size distribution of Lipo-IL11, Mn@Lipo-IL11, and Cis/Mn@Lipo-IL11. (D) TEM images of Lipo-IL11, MnO_2_, and Cis/Mn@Lipo-IL11. (E and F) EDX and elemental mapping analysis of Cis/Mn@Lipo-IL11. (G) Macroscopic images and SEM images of 2%PAMB/1%PEG-SG and 3%PAMB/1.5%PEG-SG hydrogels with Cis/Mn@Lipo-IL11+NH. (H–I) Rheological properties of 3%PAMB/1.5%PEG-SG hydrogels. (J) Changes in shear viscosity with increasing shear rate of 3%PAMB/1.5%PEG-SG hydrogels. (K) Impact of hydrogel concentration on DPPH clearance rate after 60 min. (L) Degradation rate of hydrogels at different H_2_O_2_ concentrations.

To enhance the enrichment of chemotherapy drugs at the tumor site and improve delivery efficiency, MnO_2_ and cisplatin were integrated into IL11Rα-targeted liposomes to obtain Cis/Mn@Lipo-IL11 nanoparticles. Fourier transform infrared (FTIR) spectroscopy (Fig. 2A and Fig. S1A) and proton nuclear magnetic resonance (^1^H NMR) spectrum (Figs. S1B and C) were used to detect the successful synthesis of Lipo-IL11 and PAMB-G-TK. In addition, high-resolution Mn 2p X-ray photoelectron spectroscopy (XPS) spectra confirmed the successful synthesis of mesoporous MnO_2_ (Fig. 2B). Dynamic light scattering (DLS) and zeta potential measurements demonstrate the relevant physical properties of MnO_2_ (Fig. S2). The average diameter of MnO_2_ is approximately 188.13 ± 9.77 nm, and the zeta potential exhibits a negative charge phenomenon. The loading of MnO_2_ in liposomes was investigated using zeta potential, and the results (Fig. S3) showed that after successful loading of MnO_2_ (−17.3 mV), the zeta potential of Mn@Lipo-IL11 decreased from 33.5 mV to 22.7 mV, while the Cis/Mn@Lipo-IL11 showed the comparable zeta potential to Mn@Lipo-IL11. Subsequently, DLS and transmission electron microscopy (TEM) were used to observe Cis/Mn@Lipo-IL11 nanoparticles, which showed regular spherical shape and relatively uniform size (average diameter of approximately 166.82 ± 6.81 nm) and increased hydrodynamic diameter compared to Lipo-IL11 (Fig. 2C and D). The encapsulation of cisplatin in Cis/Mn@Lipo-IL11 and its release in the tumor microenvironment in the presence of GSH were further validated using UV spectrum (Fig. S4). The results showed that Cis/Mn@Lipo-IL11 encapsulated 83.6 % of cisplatin. As expected, Cis/Mn@Lipo-IL11 exhibited GSH-dependent cisplatin release under simulated physiological conditions of tumor cells. After exposure to GSH, the release rate of cisplatin reached 60.7 ± 7.7 %, whereas under the condition without GSH, the release rate was only 28.1 ± 1.7 %. The elemental mapping and Energy Dispersive X-ray spectroscopy (EDX) analysis of Cis/Mn@Lipo-IL11 displayed the presence of Mn and Pt elements, indicating the uniform encapsulation of MnO_2_ and cisplatin (Fig. 2E and F).

### 3.2 Preparation and characterization of nanocomposite hydrogel

To prepare nanocomposite hydrogels, an addition reaction was carried out under mild conditions between the amino groups of PAMB-G-TK and the succinimide functionalities of 4-arm-PEG-SG, resulting in the formation of hydrogels with ROS-responsive property [44]. Then, Cis/Mn@Lipo-IL11 liposomes were encapsulated in the hydrogel to form Cis/Mn/NH@PGP. Subsequently, scanning electron microscopy (SEM) observations were conducted on 3%PAMB-G-TK/1.5%PEG-SG and 2 % PAMB-G-TK/1%PEG-SG hydrogels, confirming the formation of a three-dimensional network of the hydrogel, as well as the successful encapsulation and uniform distribution of Cis/Mn@Lipo-IL11 nanoparticles within the porous structure of the hydrogels (Fig. 2G). The pore diameter of the hydrogels in the porous structure shown in 3%PAMB-G-TK/1.5%PEG-SG was measured to be approximately 6 μm. However, the hydrogel prepared by 2 % PAMB-G-TK and 1 % 4-arm-PEG-SG presented the flabby state, with the weak plasticity after injection (Figs. S5 and S6). Subsequently, the cell viability of L929 cells treated with different hydrogel formulations was evaluated by the CCK-8 assay. The results showed that at all tested hydrogel formulations, the viability of L929 cells exceeded 90 %, demonstrating the excellent biocompatibility of the hydrogels (Fig. S7). In addition, the rheological properties of the hydrogels were investigated to verify the injectable property. As shown in Fig. 2H–J, the storage modulus (G′) values exceeded the loss modulus (G″), indicating the formation of stable hydrogels. Furthermore, as the shear rate increased, the viscosity decreased significantly, revealing good injectability of the hydrogels.

The ROS-responsive property was conducted using the 2,2-diphenyl-1-(2,4,6-trinitrophenyl) hydrazyl (DPPH) assay kit. The DPPH scavenging efficiency of the three hydrogels increased with the increased concentrations over 60 min, indicating continuous and effective DPPH clearance (Fig. 2K). We then monitored the weight loss of the hydrogel over time to investigate the degradation of 3%PAMB-G-TK/1.5%PEG-SG hydrogels induced by H_2_O_2_ (Fig. 2L). After incubation with PBS, a slight decrease in the weight of the hydrogels was observed (≈23 %), possibly due to the hydrolysis of ester bonds and gelatin matrix. However, as the concentration of H_2_O_2_ increased, the weight loss of the hydrogels gradually escalated. When incubated with 0.5 mM H_2_O_2_ at 37 °C, the mass of the hydrogel decreased significantly, with a loss of 91.97 ± 4.02 % at 18 days, indicating the ROS-responsive degradation of the hydrogels. To study the liposomes release from the hydrogels, Cy5.5-labeled Lipo-IL11 nanoparticles were loaded into the 3%PAMB-G-TK/1.5%PEG-SG hydrogels and soaked in the solution with different H_2_O_2_ concentrations. The *in vitro* release of Lipo-IL11 was obtained by measuring the absorbance at 680 nm (Fig. S8). It showed that the release percentage of Lipo-IL11 from the hydrogels was obviously increased as the increase of H_2_O_2_ concentration. When soaked into 0.5 mM H_2_O_2_ for 18 days, the cumulative release percentage of Lipo-IL11 was 90.31 ± 3.02 %, which was much higher than immersing into the PBS solution without H_2_O_2_ (31.89 ± 2.45 %), indicating more hydrogels degradation in the medium with addition of H_2_O_2_. This result aligned with the result of H_2_O_2_-induced hydrogels degradation, and strongly support the dependency of liposome release on ROS concentrations in the hydrogels. Therefore, 3%PAMB-G-TK/1.5%PEG-SG hydrogels would be the preferable candidate for the following studies according to the injectability and ROS responsiveness.

### 3.3 In vitro antitumor performance of Cis/Mn/NH@PGP

**Fig. 3:**
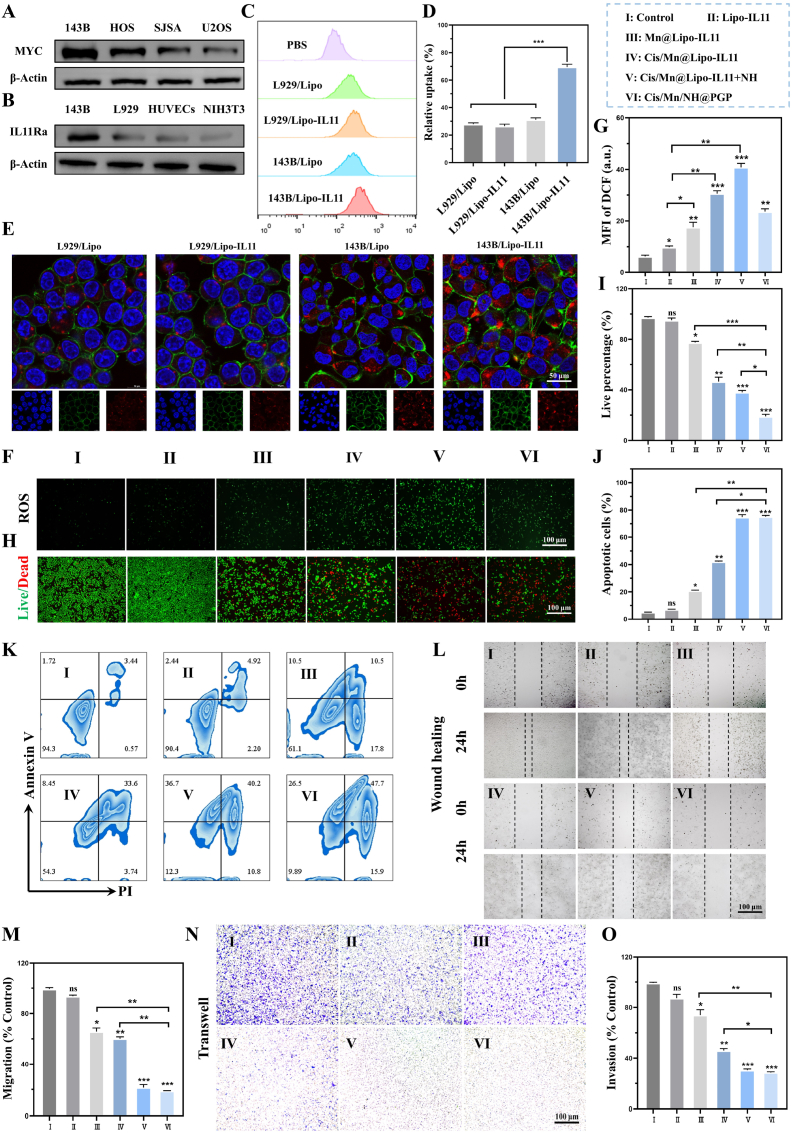
Evaluation of *in vitro* targeting ability and anti-tumor performance. (A) Detection of MYC expression in different OS cells. (B) IL11Rα expression in tumor and normal cells. (C and D) Cellular uptake of Lipo and Lipo-IL11 by L929 and 143B cells after incubation for 8 h using flow cytometry, including fluorescence intensity and cellular uptake percentage. (E) Representative fluorescence images of L929 and 143B cells co-incubated with Lipo and Lipo-IL11 for 8 h. The cell skeleton is labeled with FITC-labeled phalloidin (green); the cell nuclei are stained with 4′,6-Diamidino-2′-phenylindole (DAPI blue), and the red spots indicate the Cy5.5-labeled nanoparticles. (F, G) Representative fluorescent images and quantitative analysis of DCFH-DA staining in 143B cells after treatment for 24 h. (H, I) Live/dead staining of 143B cells after treatment for 24 h using Calcein-AM/PI kit. (J, K) Cell apoptosis analysis of 143B cells after treatment for 24 h by flow cytometry using Annexin V-FITC/PI kit. (L, M) *In vitro* migration evaluation of 143B cells after treatment for 24 h by wound healing assay. (N, O) *In vitro* invasion evaluation of 143B cells after treatment for 24 h by transwell assays. ns: no significance, ∗P < 0.05, ∗∗P < 0.01, ∗∗∗P < 0.001, compared with Control group. Ⅰ: Control, Ⅱ: Lipo-IL11, Ⅲ: Mn@Lipo-IL11, Ⅳ: Cis/Mn@Lipo-IL11, Ⅴ: Cis/Mn@Lipo-IL11+NH, Ⅵ: Cis/Mn/NH@PGP.

Firstly, western blotting was conducted to detect the expression levels of MYC and IL11Rα in different cell lines. The results showed that MYC expression was highest in 143B cells, moderate in HOS cells, and relatively low in SJSA and U2OS cells among the tumor cells (Fig. 3A). To obtain further evidence, we used western blotting to detect the differences of MYC expression between 143B cells and normal cells, including L929, HUVECs, and NIH3T3. The results showed that MYC expression was significantly higher in 143B cells, while its expression was very low in the normal cells. (Fig. S9). Furthermore, it was detected that IL11Rα expression was very high in human 143B OS cells, followed by the L929, HUVECs and NIH3T3 cells (Fig. 3B). Based on the above finding, 143B cells (high expression of IL11Rα) and L929 cells (low expression of IL11Rα) were used to validate the targeting specificity of Lipo-IL11 through flow cytometry. The results showed no significant difference in cellular uptake between Lipo and Lipo-IL11 in L929 cells, while the internalization of Lipo-IL11 in 143B cells was 69.57 ± 2.67 %, which was 2.5 times higher than that of Lipo internalized by 143B cells, suggesting good target ability of Lipo-IL11 to 143B cells (Fig. 3C and D). Additionally, the targeting ability of Lipo-IL11 was also confirmed by confocal observation using 143B cells and L929 cells. After incubation for 8 h, significant red fluorescence signals were observed in 143B and L929 cells (Fig. 3E), indicating successful cellular uptake of these nanoparticles. However, it observed that the internalization of both Lipo and Lipo-IL11 was more obvious in 143B cells than that in L929 cells. Notably, the distribution of Lipo-IL11 within the cytoplasm of 143B cells was the most, suggesting the better cellular uptake of Lipo-IL11 by 143B cells. Therefore, the result demonstrated the excellent targeting specificity of Lipo-IL11 towards 143B cells (high expression of MYC). In addition, we used western blotting to detect the differences in IL-11Rα expression in 143B cells after treatment with Lipo and Lipo-IL11. The results showed a mild downregulation of IL-11Rα expression by Lipo-IL11, which is consistent with the selective binding and killing of IL-11Rα-overexpressing tumor cells (Fig. S10).

To assess the ability of Cis/Mn@Lipo-IL11 to generate ROS *in vitro*, intracellular ROS levels were observed using the ROS fluorescent indicator 2′,7′-dichlorofluorescin diacetate (DCFH-DA) staining and analyzed by the fluorescence intensity (Fig. 3F and G). Compared to the control group, the Lipo-IL11 group showed a slight increase in the green fluorescent signal. In terms of the Mn@Lipo-IL11 group, visible increase of ROS level was occurred because of the Fenton-like reaction of incorporated MnO_2_, resulting in the production of ·OH. In contrast, in the Cis/Mn@Lipo-IL11 and Cis/Mn@Lipo-IL11+NH groups, cisplatin and Mn^2+^ bonded together to induce higher oxidative stress, leading to a significant increase in green fluorescence in the cells after treatment. The Cis/Mn/NH@PGP shows a lower ROS level compared to Cis/Mn/@Lipo-IL11 and Cis/Mn/@Lipo-IL11+NH, which can be attributed to the consumption of ROS by the TK bonds within the PGP hydrogel. Subsequently, the *in vitro* therapeutic effect of different materials against 143B cells was investigated using CCK-8 assay and Calcein-AM/PI staining experiment. The cell viability results indicated that the groups treated with Cis/Mn@Lipo-IL11, Cis/Mn@Lipo-IL11+NH, and Cis/Mn/NH@PGP had lower viability compared to other groups (Fig. S11). There was no significant difference between the Lipo-IL11 and control groups, while the Mn@Lipo-IL11 group exhibited a slight decrease in viability due to chemodynamic effect of MnO_2_. However, the increased PI-positive staining and decreased Calcein-AM-positive staining were observed for both Cis/Mn@Lipo-IL11+NH and Cis/Mn/NH@PGP groups (Fig. 3H). It can be concluded that more dead cells would be triggered by those materials. Accordingly, obviously decreased percentages of live cells could be seen from those groups (Fig. 3I). This result suggests that both Cis/Mn@Lipo-IL11+NH and Cis/Mn/NH@PGP groups could induce a more significant cytotoxic effect compared to other groups, demonstrating their enhanced therapeutic efficacy. Additionally, flow cytometry was used to detect the apoptosis rate (including early apoptosis and late apoptosis) of 143B cells cultured under different conditions. After treatment with Cis/Mn/NH@PGP, the apoptosis rate of 143B cells reached to 74.2 ± 1.6 %, which was significantly higher than the cells cultured with Cis/Mn@Lipo-IL11 (41.7 ± 0.3 %) and Mn@Lipo-IL11 (21.1 ± 0.2 %) (Fig. 3J and K). As a result, the *in vitro* anti-tumor performance of Cis/Mn/NH@PGP against 143B cells was evident. However, the Control group (untreated cells) and Lipo-IL11 group did not show high percentages of apoptosis, with the apoptotic cells of 5.4 ± 0.2 % and 7.7 ± 0.4 % respectively.

To further analyze the potential mechanism of synergistic killing effect of OS cells mediated by Cis/Mn/NH@PGP, mRNA transcriptome analysis was conducted to assess the differences in gene expression (Fig. S12). Based on volcano plots and heatmaps (p < 0.05, |fold change| ≥ 1.2), approximately 1495 differentially expressed genes (DEGs) were identified in the synergistic treatment group compared to the control group, including 499 upregulated genes and 996 downregulated genes, mainly related to the inflammation regulation and oxidative stress response. KEGG pathway enrichment analysis showed that the affected proteins primarily participated in autophagy-related pathways, including downregulation of cancer pathways such as JAK-STAT3, mTOR, cAMP and PI3K-Akt, among others (Figs. S12A–C). Next, immunoblotting experiments were performed to validate the consistent changes in representative intracellular proteins such as STAT3 (Fig. S13). Given MYC's critical role in tumor metastasis [[45], [46], [47]], Cis/Mn/NH@PGP holds promise in effectively inhibiting cancer cell invasion and metastasis. The results demonstrated that the treatment groups containing NHWD-870 led to a significant downregulation of MYC protein expression in 143B cells (Fig. S14), thereby significantly inhibiting migration and invasion against 143B cells as confirmed by the wound healing and transwell migration assays (Fig. 3L–O). These findings suggest that Mn@Lipo-IL11 inhibits OS cell migration through Mn^2+^-mediated chemodynamic therapy, while the combination of Cis/Mn@Lipo-IL11 with NHWD-870 intensifies oxidative damage to tumor cells [41,48], resulting in a synergistic effect of chemodynamic therapy-chemotherapy that significantly suppresses OS cell invasion and metastasis.

### 3.4 In vitro immune activation of Cis/Mn/NH@PGP

**Fig. 4:**
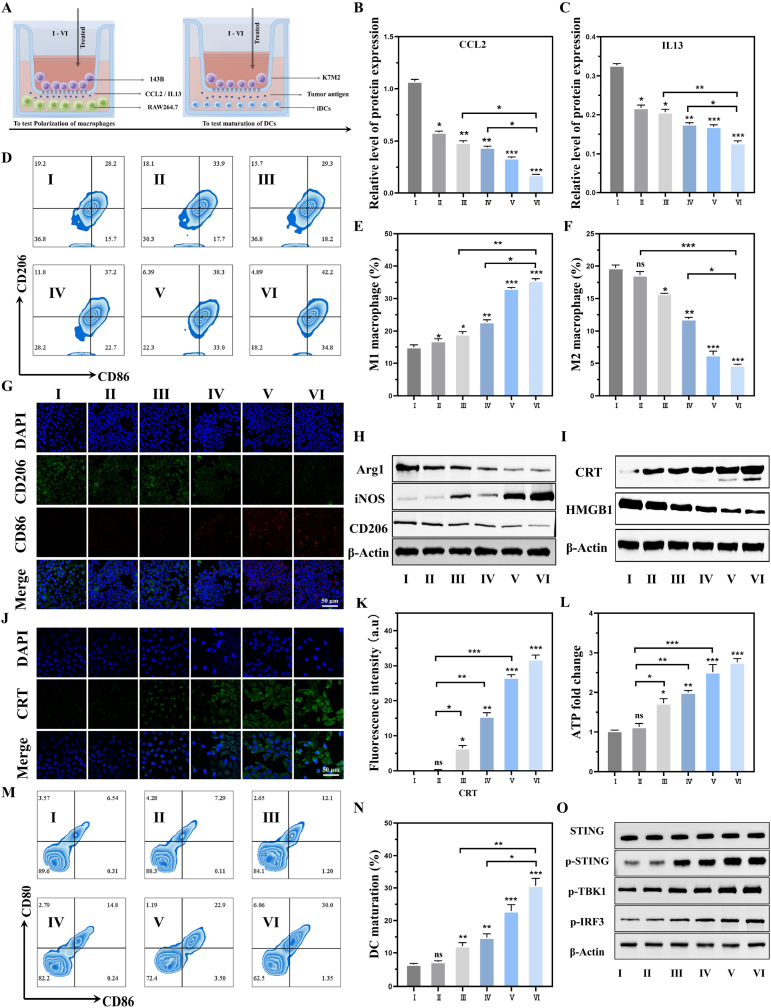
Evaluation of *in vitro* macrophage polarization and ICD induction. (A) Schematic image of the co-culture system of 143B cells with RAW264.7 and DCs (created by Figdraw). The 143B cells are seeded in the upper chamber, while RAW264.7 and DCs are seeded in the lower chamber. (B, C) ELISA analysis of CCL2 and IL13 expression levels in 143B cells. (D–F) Representative flow cytometry plots and positive percentage of M1-type macrophages (CD86^+^) and M2-type macrophages (CD206^+^). (G) Immunofluorescence images of RAW264.7 cells showing M2-type macrophages (CD206^+^, green), M1-type macrophages (CD86^+^, red), and cell nuclei (DAPI, blue). (H) Western blotting of Arg-1, CD206, and iNOS in RAW264.7 cells. (I) Western blotting of CRT and HMGB1 in K7M2 cells. (J) Immunofluorescence images of CRT (green) in K7M2 cells. (K) Quantification of fluorescence signals of CRT staining in K7M2 cells. (L) Extracellular ATP levels in K7M2 cells. (M, N) Representative flow cytometry plots and positive percentage of mature DCs (CD80^+^/CD86^+^). (O) Western blotting of the markers of Stimulator of interferon genes (STING) pathway activation in DCs. ns: no significance, ∗P < 0.05, ∗∗P < 0.01, ∗∗∗P < 0.001, compared with Control group. Ⅰ: Control, Ⅱ: Lipo-IL11, Ⅲ: Mn@Lipo-IL11, Ⅳ: Cis/Mn@Lipo-IL11, Ⅴ: Cis/Mn@Lipo-IL11+NH, Ⅵ: Cis/Mn/NH@PGP.

The Cis/Mn/NH@PGP can inhibit the function of MYC, leading to a reduction in the expression of various cell factors such as CCL2 and IL-13 in tumor cells, thereby inducing M2-type macrophages to transform into M1-type macrophages [21,49]. Calreticulin (CRT), high mobility group box 1 (HMGB1), and adenosine triphosphate (ATP), among other DAMPs, can provide auxiliary stimulation for DC cells [23,50]. Therefore, mouse monocyte macrophages (RAW264.7) and DCs were co-cultured with 143B and K7M2 cells treated with different groups in the transwell system for 24 h to assess macrophage polarization and dendritic cell maturation (Fig. 4A). First, ELISA was used to detect the secretion of CCL2 and IL13 in the supernatant of 143B cells culture. In the transwell system, Mn^2+^ and cisplatin inhibited the viability of 143B cells, subsequently reducing the secretion of pro-tumorigenic cytokines CCL2 and IL13 by these cells [21]. Compared to other groups, the levels of the pro-tumorigenic cytokines CCL2 and IL13 were significantly reduced after treatment with NHWD-870-loaded groups, especially in the Cis/Mn/NH@PGP group (Fig. 4B and C). Next, flow cytometry was used to detect the expression of CD206 (M2-type macrophage marker) and CD86 (M1-type macrophage marker) in RAW264.7 cells (the gating strategy is shown in Fig. S15). It showed that the percentage of M1 macrophages increased from 15.7 ± 0.6 % (Control group) to 35.3 ± 1.7 % (Cis/Mn/NH@PGP), while the percentage of M2 macrophages decreased to approximately one-fourth of the Control group (Fig. 4D–F). Additionally, RT-qPCR was performed to further verify the polarization of RAW264.7 (Fig. S16). It can be observed that the macrophages in the groups containing NHWD-870 (Cis/Mn@Lipo-IL11+NH and Cis/Mn/NH@PGP) showed significantly increased expression of M1 markers (iNOS) and decreased expression of M2 markers (Arg-1 and CD206), indicating successful reprogramming of macrophages. While treatments with NHWD-870 increased M1 marker expression and reduced M2 markers, macrophage polarization was also observed in controls without NHWD-870. This suggests that other components, such as Mn^2+^ or cisplatin, may independently drive M1 polarization, consistent with previous reports [51]. To obtain further evidence, immunofluorescence staining of CD86 and CD206) was conducted for validation, as shown in Fig. 4G. Strong red fluorescence signals for CD86 were detected in both the Cis/Mn@Lipo-IL11+NH and Cis/Mn/NH@PGP groups while either undetectable or weakly expression in the PBS, Lipo-IL11, Mn@Lipo-IL11, and Cis/Mn@Lipo-IL11 groups was observed. In contrast, the expression of CD206 (green fluorescence signals) in RAW264.7 showed the opposite tendency. Also, the western blotting results showed the downregulation of CD206 and Arg-1 and upregulation of iNOS (Fig. 4H). These results indicate that Cis/Mn/NH@PGP can promote the reprogramming of macrophage phenotype.

Next, the enhancement of ICD induced by Cis/Mn/NH@PGP and its promotion of DCs maturation were examined. Compared to the Control and Lipo-IL11 groups, CRT expression in K7M2 cells was significantly upregulated in the Mn@Lipo-IL11, Cis/Mn@Lipo-IL11, Cis/Mn@Lipo-IL11+NH, and Cis/Mn/NH@PGP groups (Fig. 4I). In contrast, HMGB1 expression levels were decreased in those groups, with the most significant reduction in the Cis/Mn@Lipo-IL11+NH and Cis/Mn/NH@PGP groups. Immunofluorescence staining of CRT was performed to observe CRT expression in K7M2 cells (Fig. 4J and K). The cells in the Control group and the Lipo-IL11 group showed minimal CRT signals, whereas the cells in the Mn@Lipo-IL11, Cis/Mn@Lipo-IL11, Cis/Mn@Lipo-IL11+NH, and Cis/Mn/NH@PGP groups exhibited significant CRT staining signals. As for the ELISA of HMGB1, compared to the control group, the addition of MnO_2_ and cisplatin significantly increased the expression of HMGB1 released by K7M2 (Fig. S17). We further validated the extracellular level of ATP from K7M2 cells after different treatments. For the Mn@Lipo-IL11 and Cis/Mn@Lipo-IL11 groups, the extracellular levels of ATP were 1.6 times and 1.9 times higher than that of the Control group. In terms of the Cis/Mn@Lipo-IL11+NH and Cis/Mn/NH@PGP groups, the extracellular ATP levels were approximately 2.5 times and 2.7 times higher than that in the Control group, suggesting the amplified ICD process which was associated with the stimulatory roles of encapsulated cisplatin and Mn ions [52,53]. As for the treatment with Lipo-IL11 alone, it did not affect extracellular ATP level (Fig. 4L). These results suggest that the Cis/Mn/NH@PGP group can induce a stronger ICD effect through the synergistic action of CRT exposure, HMGB1 release, and ATP secretion.

It is considered that typical biomarkers of ICD, molecules such as ATP, CRT, and HMGB1 can induce dendritic cell maturation and activate cytotoxic T lymphocytes, thereby promoting immune responses [20,54]. Thus, the number of mature DCs (CD80^+^ and CD86^+^ cells) after different treatment was measured by flow cytometry. The proportion of mature cells significantly increased from 6.49 ± 0.46 % (Control group) to 30.7 ± 3.35 % (Cis/Mn/NH@PGP group) (Fig. 4M and N). As shown in Fig. 4O, the Cis/Mn/NH@PGP treatment significantly upregulated the phosphorylation of STING, TANK binding kinase 1 (TBK1), and interferon regulatory factor 3 (IRF3) in DCs, indicating the activation of the STING pathway. Additionally, previous study has reported the role of Mn^2+^ in activating the STING pathway [55,56]. Thus, the Mn^2+^ released from Cis/Mn/NH@PGP probably played a key role in this activation by enhancing the cGAS-STING-mediated immune response. These results demonstrate that Cis/Mn/NH@PGP can promote the maturation of DCs by inducing ICD through the synergistic effects of Mn^2+^ and other components, thereby enhancing the antitumor immune response.

### 3.5 In vivo antitumor performance of Cis/Mn/NH@PGP

**Fig. 5:**
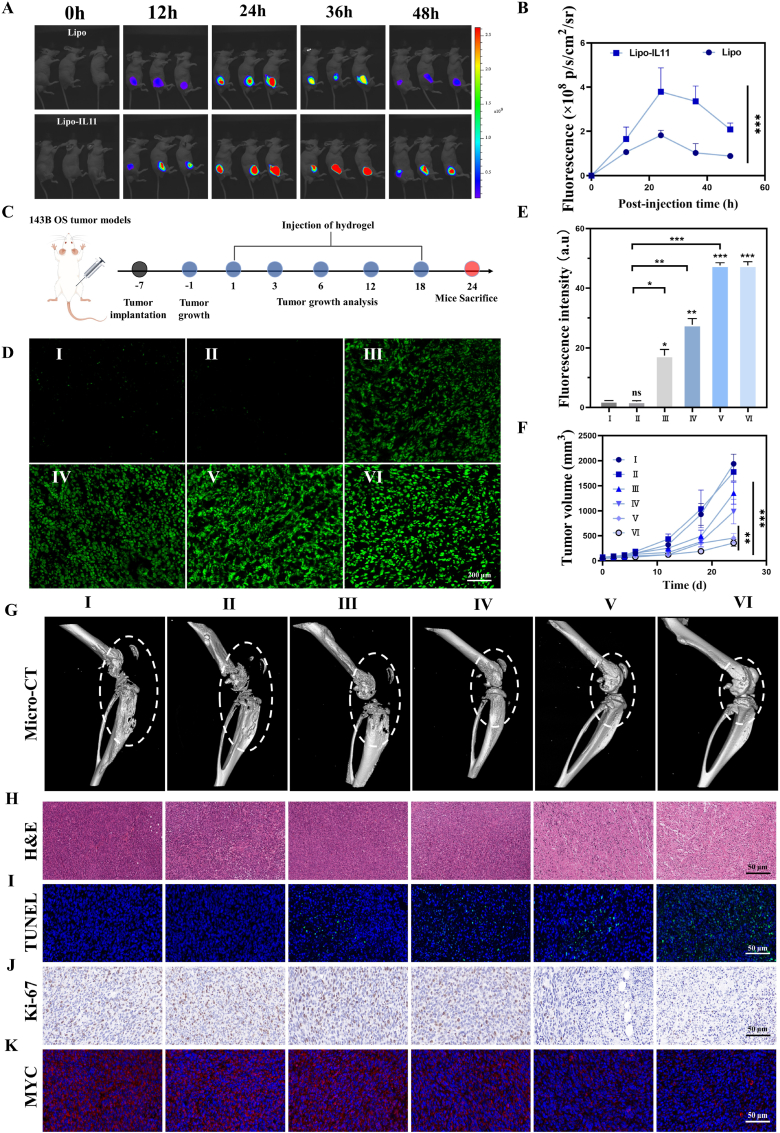
Evaluation of *in vivo* antitumor performance in 143B-bearing OS model using injectable hydrogels. (A, B) Representative fluorescence images and quantitative analysis of fluorescence signal intensity in mice after intravenous injection with Lipo and Lipo-IL11 (labeled with Cy5.5). (C) Schematic image showing the treatment of orthotopic 143B-bearing OS model in mice (created by Figdraw). (D, E) Representative fluorescence images and fluorescence intensity analysis of ROS levels in tumor. (F) Assessment of tumor volume changes in each group after different treatment. (G) Reconstructed micro-CT images of joints in tumor-bearing mice (white dashed lines indicate bone destruction in tumor areas). (H–J) Representative images of H&E staining, immunofluorescence staining of TUNEL and immunohistochemical staining of Ki-67 in tumors. (K) Representative images of immunofluorescence staining of MYC in tumor. ns: no significance, ∗P < 0.05, ∗∗P < 0.01, ∗∗∗P < 0.001, compared with Control group. Ⅰ: Control, Ⅱ: PGP, Ⅲ: Mn@PGP, Ⅳ: Cis/Mn@PGP, Ⅴ: Mn/NH@PGP, Ⅵ: Cis/Mn/NH@PGP.

The therapeutic efficacy of Cis/Mn/NH@PGP was evaluated in an orthotopic tumor model in nude mice. The orthotopic 143B-bearing tumor model was established by injection of 143B cells into the tibia of Balb/c nude mice. An *in vivo* imaging system (IVIS) was used to study the accumulation and biodistribution of Lipo and Lipo-IL11 in tumor sites after intravenous administration. After injection of liposomes, the fluorescence signal in all mice tumor sites gradually increased and reached its maximum at 24 h, while the tumor accumulation in the Lipo group was much lower compared to the Lipo-IL11 group (Fig. 5A and B). Afterward, we systematically monitored the biodistribution of the liposomes during the treatment process. *In vitro* imaging at 24 h showed a high accumulation of Lipo-IL11 in the tumor, while low accumulation was observed in the major organs (Fig. S18). These results revealed the important role of IL11 peptide modification in promoting Lipo-IL11 accumulation in 143B OS tumors.

According to the treatment scheme shown in Fig. 5C, the therapeutic efficacy of Cis/Mn/NH@PGP *in vivo* was evaluated using a 143B-bearing OS model through tumor size and bone destruction. To validate the generation of ROS by hydrogels, the ROS fluorescence signals were detected in tumor specimens (Fig. 5D). In tumors injected with PGP hydrogels, only weak ROS fluorescence signals were detected, similar to the Control group. In contrast, significant ROS fluorescence signals were observed in tumors injected with Mn@PGP, demonstrating the generation of ROS mediated by Mn^2+^ through the Fenton-like reaction. The fluorescence intensity of ROS generation in Cis/Mn@PGP group was obviously increased because of the Fenton-like reaction and oxidative stress induced by cisplatin, whereas the Cis/Mn/NH@PGP group with the encapsulation of NHWD-870 presented a higher degree of ROS generation (Fig. 5E). Tumor growth was monitored by tumor volume measurement over the 24-day period to assess antitumor efficacy (Fig. 5F). As shown in the growth curves of different treatments, the tumor growth in the Control and PGP groups was nearly identical and more prominent. Compared with those groups, the tumor growth trends in other groups were obviously attenuated, especially for Mn/NH@PGP and Cis/Mn/NH@PGP groups. The Mn/NH@PGP group displayed a significant reduction in tumor volume, suggesting the inhibitory effect on tumor growth, which was probably due to the chemodynamic activity of Mn^2+^ and the NHWD-870-mediated modulation of tumor-associated pathways. It was noted that the Cis/Mn/NH@PGP group showed the most significant inhibition of tumor growth, with the smallest tumor volume at 24 days. Furthermore, the photographs of tumor-bearing tibia of the mice taken after treatment also supported the above results (Fig. S19), showing the smallest size of tumor specimens for the Cis/Mn/NH@PGP group. We used micro-computed tomography (micro-CT) to collect images of the tumor tibia and evaluate its inhibitory effect on bone destruction. Reconstructed images showed that the tibia of the Cis/Mn/NH@PGP group had the greatest integrity as compared to other groups (Fig. 5G), which may be related to the group's superior antitumor efficacy. Through Hematoxylin-eosin (H&E) staining, Terminal Deoxynucleotidyl Transferase-Mediated dUTP Nick End Labeling (TUNEL) staining, and Ki-67 staining of the tumor tissue, we found that the Cis/Mn/NH@PGP group exhibited the strongest damage to the tumor tissue, with significant inhibition on tumor cell proliferation and significant enhancement on apoptosis (Fig. 5H–J). To obtain further information on the targeted degradation ability of MYC by these nanocomposite hydrogels, tumor tissue was collected and treated with immunofluorescence staining of MYC at 24 days after treatment, which demonstrated significant inhibition of MYC protein after treatment with Cis/Mn/NH@PGP (Fig. 5K). In addition, we performed immunofluorescence staining to analyze IL11Rα expression levels in tumor tissues and normal bone tissues after treatment (Fig. S20). Obviously, high level of IL11Rα expression in the tumor tissue without treatment could be observed. In contrast, IL11Rα expression remained relatively low in normal bone tissues. After treatment with Cis/Mn/NH@PGP, IL11Rα expression in the tumor tissues was reduced. This result reveals that the IL11Rα expression is predominantly tumor-specific and the Cis/Mn/NH@PGP treatment can induce tumor cell death. Although the reduced IL11Rα expression may affect the targeting efficacy to tumor site, a higher IL11Rα expression in tumor tissue compared to that of normal tissues was still dominant and could enhance the therapeutic efficacy. These results suggest that the Cis/Mn/NH@PGP hydrogel can increase ROS generation, promote apoptosis, inhibit MYC expression, and thereby suppressing tumor growth and bone destruction.

To further evaluate the *in vivo* biosafety of Cis/Mn/NH@PGP hydrogel, the body weight of mice under different treatments was monitored. The result showed the stable changes of body weight, even at the end of treatment, indicating good biosecurity of these hydrogels (Fig. S21). Additionally, the effect of injectable hydrogels on major organs in mice was assessed by H&E staining (Fig. S22). It did not show obvious changes of the hydrogel groups compared with the Control group. Also, the levels of various blood biochemical markers in each group were quantified through blood biochemical analysis (Fig. S23). There were no significant differences observed between the Control group and the treatment groups in terms of major blood biochemical markers (RBC, MCH, PDW, HGB and WBC). These findings support the apparent biosafety of the antitumor process based on the nanocomposite hydrogels. Additionally, we also assessed the degradation of Cis/Mn/NH@PGP *in vivo*. After subcutaneous implantation for 7 days, obvious degradation was observed and the residual content of Cis/Mn/NH@PGP hydrogel was 42.7 ± 3.1 %, demonstrating its degradable property (Fig. S24).

### 3.6 In vivo immunomodulatory performance of Cis/Mn/NH@PGP

**Fig. 6:**
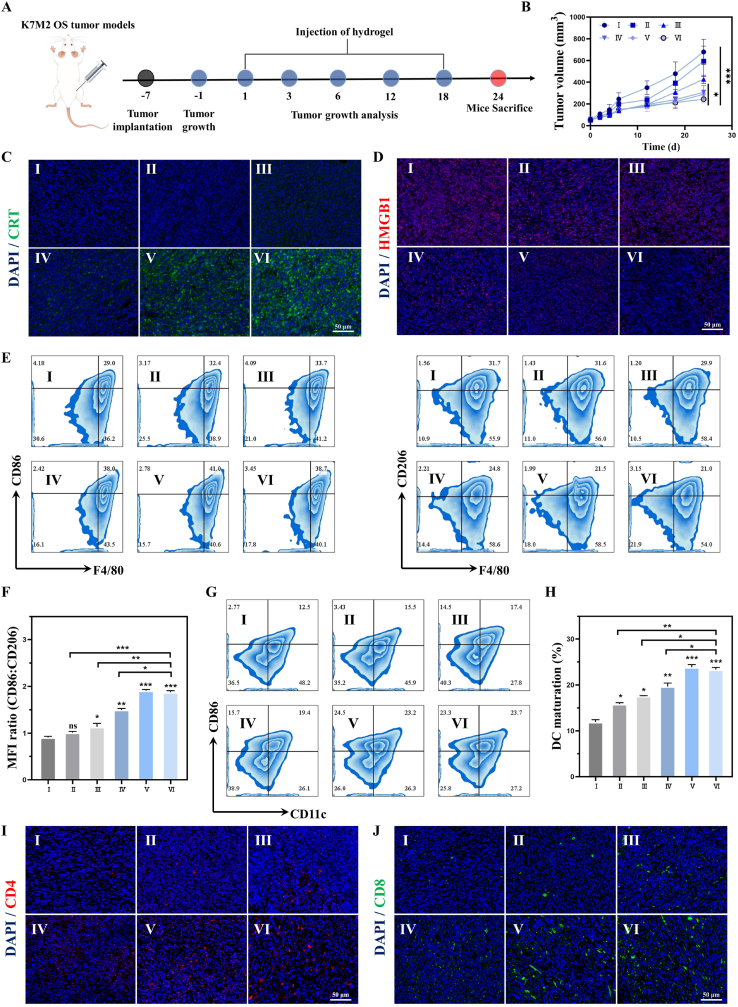
Evaluation of *in vivo* antitumor performance in subcutaneous K7M2-bearing OS model using injectable hydrogels. (A) Schematic image showing the treatment of subcutaneous K7M2-bearing OS model in mice (created by Figdraw). (B) Measurement of tumor volume changes in each group after different treatment. (C) Immunofluorescence images of CRT in tumor. (D) Immunofluorescence images of HMGB1 in tumor. (E, F) Representative flow cytometry plots and quantitative analysis of M1 marker expression (F4/80^+^/CD86^+^) and M2 marker expression (F4/80^+^/CD206^+^) and ratio of CD86^+^/CD206^+^. (G, H) Representative flow cytometry plots and quantitative analysis of mature DCs. (I, J) Immunofluorescence staining of CD4^+^ and CD8^+^ cells in tumor. ns: no significance, ∗P < 0.05, ∗∗P < 0.01, ∗∗∗P < 0.001, compared with Control group. Ⅰ: Control, Ⅱ: PGP, Ⅲ: Mn@PGP, Ⅳ: Cis/Mn@PGP, Ⅴ: Mn/NH@PGP, Ⅵ: Cis/Mn/NH@PGP.

To evaluate the immunomodulatory therapy of Cis/Mn/NH@PGP hydrogel on subcutaneous OS tumor of mice, K7M2 cells were injected subcutaneously into Balb/c mice. The experimental procedure was performed as shown in Fig. 6A. The antitumor efficacy was studied by assessing tumor growth in terms of the tumor volume, tumor weight, histological staining and so on. As expected, the Cis/Mn/NH@PGP group exhibited the smallest tumor volume and weight after treatment (Fig. 6B, Figs. S25 and S26). The H&E staining of tumor sections revealed a significant decrease in nuclear density of tumor cells in the Mn/NH@PGP group and Cis/Mn/NH@PGP group. TUNEL staining showed intense green fluorescence observed in tumor tissues treated with Cis/Mn/NH@PGP (Fig. S27), demonstrating the promotive effect on apoptosis of tumor cells. Subsequently, tumor tissues were collected to detect the expression of CCL2 and IL13 using ELISA (Fig. S28). The most significant decrease in CCL2 and IL13 expression was observed in Cis/Mn/NH@PGP groups. Specifically, the expression levels of CCL2 and IL13 in Cis/Mn/NH@PGP group were reduced by 76.3 % and 73.1 % as compared to the Control group, respectively. We found that treatment with Cis/Mn/NH@PGP significantly enhanced the induction effect of ICD in tumors. The ATP content in the PGP group was similar to that in the Control group, while the ATP content in the tumors of the Cis/Mn/NH@PGP group increased by 4.8-fold (Fig. S29). As shown in the fluorescence staining images of CRT, the Cis/Mn/NH@PGP group exhibits the strongest fluorescence signal, validating the highest level of CRT (Fig. 6C). The fluorescence intensity of CRT staining of the Mn/NH@PGP and Cis/Mn@PGP groups was significantly higher than that of the Mn@PGP and PGP groups, while the Cis/Mn/NH@PGP group showed the highest degree of fluorescence intensity (Fig. S30A). Compared to the Control and PGP groups, the HMGB1 staining is significantly reduced in the Mn@PGP, Cis/Mn@PGP, Mn/NH@PGP and Cis/Mn/NH@PGP groups (Fig. 6D). The results demonstrate that the observed increase in ATP levels is consistent with ICD induction, wherein extracellular ATP promotes dendritic cell activation. Conversely, the decrease in HMGB1 levels *in vivo* may be attributed to its rapid binding to extracellular matrix components or uptake by immune cells, reflecting its dynamic regulation within the tumor microenvironment [23]. Among them, the levels of HMGB1 in the Mn/NH@PGP and Cis/Mn/NH@PGP groups are the lowest, indicating cellular secretion of HMGB1(Fig. S30B). These results confirmed that Cis/Mn/NH@PGP could induce augmented ICD for immune response activation.

Next, various immune cells in the obtained tumor tissue were investigated. After treatment with Cis/Mn/NH@PGP, the ratio of M1-like macrophages to M2-like macrophages and the percentage of mature DCs significantly increased, as determined by the results of flow cytometry analysis (Fig. 6E–H and Fig. S31). This indicates the successful activation of the immune response by Cis/Mn/NH@PGP. The generated M1-like macrophages and mature DCs further activate T cells for tumor immunotherapy, resulting in a significant increase in the ratio of helper T cells (CD4^+^) and cytotoxic T cells (CD8^+^) after administration of Cis/Mn/NH@PGP (Fig. S32). The increment of the Cis/Mn/NH@PGP group is higher than that of the Cis/Mn@PGP group, which may be attributed to the additional contribution of MYC degradation to immunotherapy. Furthermore, the enhanced immunofluorescence was observed in tumor tissue after treatment with Cis/Mn/NH@PGP, indicating the significant increase of tumor-infiltrating CD4^+^ T cells and CD8^+^ T cells (Fig. 6I and J). As a result, the Cis/Mn/NH@PGP exhibits significant inhibition effect on MYC and promotion on ICD level, thereby resulting in obvious infiltration of T cells for immunotherapy in tumor tissues.

### 3.7 In vivo immune infiltration and anti-metastatic capability of Cis/Mn/NH@PGP

**Fig. 7:**
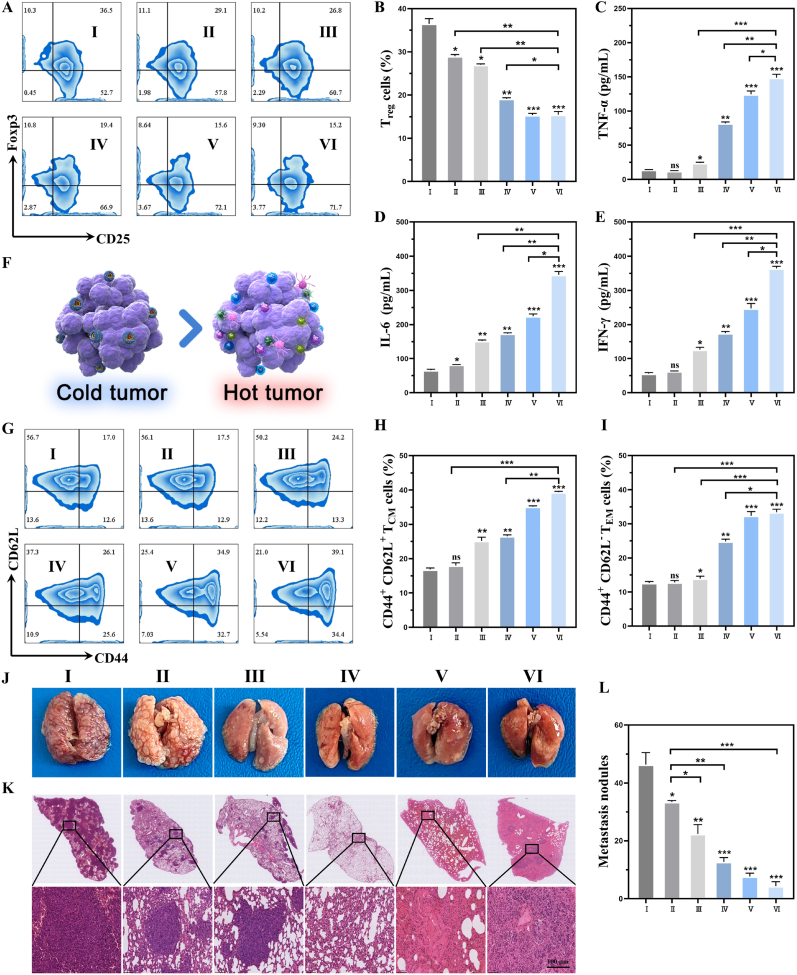
Evaluation of immune response activation and lung metastasis inhibition in subcutaneous K7M2-bearing OS model using injectable hydrogels. (A) Representative flow cytometry plots of tumor-infiltrating Treg cells in tumor. (B) Quantitative analysis of tumor-infiltrating Treg cells. (C–E) Secretion levels of pro-inflammatory cytokines (TNF-α, IL-6 and IFN-γ) in serum. (F) Schematic illustration showing the enhanced immune infiltration from cold tumor to hot tumor. (G–I) Representative flow cytometry plots and quantitative analysis of CD44^+^CD62L^+^ TCM cells and CD44^+^CD62L^−^ TEM cells in spleen. (J) Representative images of nodules in metastatic lung. (K) H&E staining of lung tissue. (L) Quantitative analysis of nodules in metastatic lung. ns: no significance, ∗P < 0.05, ∗∗P < 0.01, ∗∗∗P < 0.001, compared with Control group. Ⅰ: Control, Ⅱ: PGP, Ⅲ: Mn@PGP, Ⅳ: Cis/Mn@PGP, Ⅴ: Mn/NH@PGP, Ⅵ: Cis/Mn/NH@PGP.

After treatment with different hydrogels, the quantity of regulatory T (Treg) cells, an essential immune-suppressive cell, was evaluated in the tumor microenvironment. The Treg cell levels in the Cis/Mn@PGP and Cis/Mn/NH@PGP groups were reduced to 19.2 ± 0.4 % and 15.4 ± 0.4 %, respectively, due to the promoting effect of NHWD-870 release and the ICD induced by Mn^2+^ and cisplatin [53]. This reduction of Treg cell levels contributes to reversing the immune-suppressive microenvironment. (Fig. 7A and B). Meanwhile, it was observed that the secretion of pro-inflammatory cytokines (including Tumor necrosis factor-alpha (TNF-α), Interleukin-6 (IL-6), and Interferon-γ (IFN-γ)) in the Cis/Mn/NH@PGP treatment group was higher than that in the Mn@PGP group and the Cis/Mn@PGP group (Fig. 7C–E). This indicates that the degradation of MYC and the release of ICD factors enhanced tumor immune infiltration and subsequent immune activation. As illustrated in Fig. 7F, the outcomes of Cis/Mn/NH@PGP transformed the immunosuppressive “cold” tumors into immunostimulatory “hot” tumors. Encouraged by the practical antitumor effects and enhanced tumor immune response of Cis/Mn/NH@PGP, we examined whether our strategy could prevent systemic tumor metastasis in the tumor model by generating immunological memory effects. In this study, flow cytometry was conducted to assess the proportion of memory T cells in the spleens after treatment for 24 days. As shown in Fig. 7G–I, the proportions of T_CM_ and T_EM_ cells in Cis/Mn/NH@PGP group were significantly higher than those in the other groups. The results demonstrate that the immune-suppressive microenvironment of the tumor has been reshaped, establishing a persistent immune memory effect.

Furthermore, the tumor metastasis was assessed after treatment with different hydrogels. By monitoring the lungs of mice carrying K7M2 tumors for 24 days, it revealed that mice treated with Cis/Mn@PGP, Mn/NH@PGP and Cis/Mn/NH@PGP showed significantly fewer cancerous nodules compared to the Control and PGP treatment (Fig. 7J). H&E staining and quantitative analysis of nodules on lung metastases further indicated a significant reduction in the degree of lung metastasis of tumors following treatment with Cis/Mn/NH@PGP (Fig. 7K and L). This result suggests that treatment with Cis/Mn/NH@PGP can generate a stronger memory immune effect, facilitating the establishment of active immune responses and preventing lung metastasis for OS development.

The MYC oncogene is one of the most commonly activated oncogenes in human cancers. It primarily functions as a transcription factor, directly or indirectly regulating the expression of thousands of genes to promote tumor growth. Additionally, MYC exerts various biological effects on cellular programs, influencing intrinsic cellular biology, the host immune system, and the tumor microenvironment [[57], [58], [59]]. Up to 70 % of cancer types exhibit dysregulated MYC gene expression. Targeting MYC directly or indirectly has shown promising potential in anti-tumor strategies. Several drugs targeting MYC have been developed, including bromodomain and extra-terminal domain (BET) inhibitors, MYCi361, and aurora kinase inhibitors [17,60,61]. However, these compounds have shown poor clinical trial efficacy, primarily due to potential off-target effects and drug resistance. Among them, NHWD-870, as a BET inhibitor, binds to acetylated histones, affects the recruitment of transcription factors, and regulates transcription. It is noteworthy that bromodomain-containing protein 4 (BRD4) can regulate MYC transcription by directly binding to the MYC promoter region. Therefore, BRD4 inhibitors are also considered as MYC inhibitors. For instance, JQ-1 carboxylic acid (JQ1) has been demonstrated to downregulate MYC expression and inhibit tumor growth in various animal models of MYC activation [14,62,63]. Previous studies have found that the small molecule drug NHWD-870 inhibits OS growth by regulating MYC levels. NHWD-870 can regulate the expression of tumor-associated macrophage colony-stimulating factor (CSF1) through HIF-1α, thereby blocking the proliferation of tumor-associated macrophages and enhancing tumor immune infiltration [10].

In this work, we designed a liposome with IL-11 modification for targeting IL11Rα that highly expressed in the OS cells, enabling enhanced membrane penetration for precise drug delivery. Our results support that this drug delivery system can effectively penetrate membranes and achieve precisely targeted destruction of cancer cells while avoiding harmful effects on normal cells. In addition, we developed a hydrogel specifically designed to target the elevated ROS concentrations within the tumor microenvironment. By administering it *via* subcutaneous injection, we were able to achieve sustained and efficient delivery of the composite nanogel system containing NHWD-870 and Cis/Mn@Lipo-IL11, a feature that sets it apart from most commercially available hydrogels used in clinical settings. The elevated levels of GSH and ROS are known to occur in many tumor microenvironments, thus the use of GSH/ROS-dependent drug release characteristics was widely accepted [64,65]. Inspired by these concepts, cisplatin-loaded MnO_2_ was encapsulated into the IL11Rα-targeted liposomes and then incorporated into the ROS-responsive injectable hydrogels with NHWD-870 loading. Benefiting from the high ROS levels in the tumor microenvironment, ROS-responsive hydrogel-based nanomedicines offer an alternative approach for local drug delivery, achieving precise and sustained drug release *via* intratumoral injection. Compared with conventional injectable hydrogels, our developed nanocomposite hydrogel possessed many advantages, including good compatibility, ROS-responsive degradation, and highly efficient encapsulation and targeted delivery of drugs into the MYC-amplified OS. The *in vivo* data support the effectiveness of this modular design in inhibiting OS tumor growth. Our findings indicate that this drug delivery strategy effectively inhibits tumor growth and metastasis through the chemodynamic therapy-chemotherapy-immunotherapy combination therapy, thereby supporting its potential for future clinical translation. Additionally, incorporating PD-1 blockade therapy could further enhance the immune response against tumors, providing a complementary approach to our strategy. By reactivating T cells and promoting their ability to attack cancer cells, PD-1 blockade therapy may improve treatment outcomes and contribute to a more robust and durable anti-tumor immunity. Although we have demonstrated significant anti-tumor efficacy of designed nanocomposite hydrogel, limitations of our study include the evaluation of effectiveness in mouse-derived models rather than patient-derived xenografts (PDX) [66,67].

## 4 Conclusion

Nanocomposite hydrogel technology offers an opportunity to target multiple vulnerabilities of OS simultaneously. In this study, we reported an injectable nanocomposite hydrogel (Cis/Mn/NH@PGP) composed of a TME-responsive polymer framework and multi-structured nanoparticles (Cis/Mn@Lipo-IL11). The results demonstrate that the Cis/Mn/NH@PGP hydrogels deliver several drugs in physiological response, providing localized, dynamic immunotherapy and chemotherapy effects for OS. In the animal models, the Cis/Mn/NH@PGP hydrogels underwent *in situ* gelation upon injection and degrades within the tumor microenvironment over time, continuously releasing NHWD-870 and embedded nanoparticles for cisplatin release, thus inhibiting OS tumor growth and metastasis by degrading MYC and modulating the immune-suppressive TME. In summary, this study presented an effective therapeutic strategy with highly targeted drug delivery efficiency and rational combination treatment for precision therapy of MYC-amplified osteosarcoma.

## CRediT authorship contribution statement

**Yichao Ma:** Writing – original draft, Methodology, Investigation, Formal analysis, Data curation. **Peng Lai:** Writing – original draft, Validation, Methodology, Formal analysis. **Zhou Sha:** Writing – original draft, Validation, Methodology, Formal analysis. **Bing Li:** Validation, Formal analysis. **Jiangpeng Wu:** Validation, Formal analysis. **Xiaojun Zhou:** Writing – review & editing, Supervision, Funding acquisition. **Chuanglong He:** Writing – review & editing, Supervision, Resources, Conceptualization. **Xiaojun Ma:** Writing – review & editing, Supervision, Resources, Project administration, Funding acquisition, Conceptualization.

## Data availability statement

The data that support the findings of this study are available from the corresponding author upon reasonable request.

## Ethics approval and consent to participate

This study does not involve human participants, human data, or human tissues. All the animal studies were conducted following the guidelines of the Institutional Animal Care and Use Committee and approved by the Ethics Committee of Shanghai General Hospital, Shanghai Jiao Tong University School of Medicine (2023SQ084).

## Declaration of competing interest

The authors declare that they have no known competing financial interests or personal relationships that could have appeared to influence the work reported in this paper.
