# Supplementary material for: TME-responsive nanocomposite hydrogel with targeted capacity for enhanced synergistic chemoimmunotherapy of MYC-amplified osteosarcoma
Source: Bioact Mater. 2025 Jan 14;47:83–99. doi: 10.1016/j.bioactmat.2025.01.006 (PMC11783017; doi:10.1016/j.bioactmat.2025.01.006)
Supplement: Multimedia component 1 [file mmc1.docx]

**S1. Additional Experimental Sections**

**Materials:** Tetraethyl orthosilicate (TEOS), potassium permanganate (KMnO_4_), ammonium hydroxide (approximately 25-28% ammonia solution), calcium chloride dihydrate (CaCl_2_·2H_2_O), and ammonium bicarbonate (ammonium hydrogen carbonate), m-xylene, ethanol, methanol, dimethyl sulfoxide (DMSO), and hydrochloric acid (HCl), 1-(3-Dimethylaminopropyl)-3-ethylcarbodiimide hydrochloride (EDC·HCl), 2,2-Diphenyl-1-picrylhydrazyl (DPPH), 3-Amino-4-methoxybenzoic acid (AMB), and ammonium persulfate (APS) were purchased from Sinopharm Chemical Reagent Co., Ltd. (Shanghai, China). 4-arm poly (ethylene glycol) succinimidyl glutarate (4-arm-PEG-SG) was obtained from Xi'an Ruixi Biological Technology Co., Ltd. Cisplatin and NHWD-870 were purchased from Macklin Biochemical Co., Ltd. (Shanghai, China). Phosphate Buffered Saline (PBS, pH 7.4) were received from Gibco Life Technologies Co. (Carlsbad, CA, USA). All cell culture agents were purchased from Life Technologies (China). All antibodies were provided by Biolegend (USA) and Abcam (USA). All assay kits were purchased from Beyotime Biotechnology (Shanghai, China) and R&D Systems (USA). All solvent was provided by Sinopharm (Shanghai, China).

**Synthesis of MnO_2_** **Nanoparticles:** By referencing previous report [1], 14 ml of ethanol, 2 ml of deionized water, and 50 μL of ammonia solution were added into to a round-bottom flask, and stirred at 50°C for 5 minutes using a magnetic stirrer. Then, 500 μL of TEOS was added dropwise into the mixture and stirred for 2 hours. Afterward, the mixture was centrifuged at a speed of 14800 rpm for 5 minutes to obtain the SiO_2_ nanoparticles. Next, to prepare MnO_2_ nanoparticles, the SiO_2_ nanoparticles were dispersed in 10 mL of deionized water, then added dropwise with a 20 mL aqueous solution containing KMnO_4_ (80 mg) under ultrasonic conditions. After the complete addition of KMnO_4_, the mixture was treated with continuous sonication for 1 hour and stirred overnight at room temperature. Subsequently, the reaction mixture was centrifuged at 14800 rpm for 5 minutes, followed by removing the supernatant to obtain SiO_2_@MnO_2_ nanoparticles. The resultant nanoparticles were dispersed into a solution of sodium carbonate (4.29 g, 20 μL) and stirred overnight at 60°C. After centrifuging the reaction at a speed of 14800 rpm for 5 minutes, the precipitate was washed with deionized water to obtain the MnO_2_ nanoparticles with hollow structure.

**Synthesis of DSPE-PEG-IL11:** First, DSPE-PEG-NHS and IL11 peptide (China Peptides Co., Ltd. QYAOBIO, Shanghai, China) were reacted to prepare the copolymer DSPE-PEG-IL11. 200 mg of DSPE-PEG-NHS was dissolved in 1 mL of N,N-dimethylformamide (DMF), and 10.58 mg of IL11 peptide was dissolved in 0.1 mL of DMF. Under nitrogen protection, the DSPE-PEG-NHS solution was slowly added to the IL11 peptide solution at 30°C for 30 minutes. After continuous stirring for 24 hours, the mixed solution was dialyzed against DMF and dichloromethane (DCM) solutions. Finally, the product DSPE-PEG-IL11 was recovered by precipitation in cold ether and vacuum drying. Fourier-transform infrared spectroscopy (FTIR) and proton nuclear magnetic resonance (^1^H NMR) were used to the synthesis of DSPE-PEG-IL11.

**Preparation of Cis/Mn@Lipo-IL11 Liposomes:** Liposomes loaded with MnO_2_ and cisplatin were prepared using the thin-film hydration method. (2,3-Dioleylphosphatidylethanolamine) triethylammonium salt (DOTAP), cholesterol, and DSPE-PEG-IL11 were dissolved in chloroform with a molar ratio of 10:10:1. Chloroform was removed by rotary evaporation to obtain a lipid film. Lipo-IL11 and Lipo with Cy5.5 labelling were prepared according to the reported procedure [2]. Subsequently, 2 mL of PBS solution containing MnO_2_ was added to the dried lipid film to obtain MnO_2_-loaded liposomes (Mn@Lipo-IL11). Then, the liposomes were extruded using a liposome extruder to obtain uniformly sized single-layer liposomes. The liposomes were freeze-dried and then mixed with cisplatin solution (30 μM). Finally, through hydration, liposomes encapsulating both MnO_2_ and cisplatin, termed as Cis/Mn@Lipo-IL11, were obtained.

**Determination of Drug Loading of Cis/Mn@Lipo-IL11:** The Cisplatin content of Cis/Mn@Lipo-IL11 was determined by UV spectrum according to the standard curve of Cisplatin [3]. Drug loading content (DLC) were calculated using the following equations:

$\text{DLC (}\text{wt}\text{\%)}\text{ = }\frac{\text{Weight of loaded Cisplatin}}{\text{Weight of polymers and loaded Cisplatin}}\text{ × }\text{100\%}$ (1)

The *in vitro* Cisplatin release behavior from Cis/Mn@Lipo-IL11 (200 μg/mL) was studied at 37℃ using a dialysis method (MWCO 14000 Da) in 25 mL PBS (pH 7.4, 10 mM) with or without 10 mM GSH under stirring (100 rpm).

**Characterization of Nanoparticles:** Malvern Zetasizer (Nano ZS90) was used to measure the hydrodynamic diameters and surface potentials of MnO_2_, Lipo-IL11、Mn@Lipo-IL11 and Cis/Mn@Lipo-IL11 (200 μg/mL). Transmission electron microscope (TEM, Tecnai G2) was employed to characterize the morphology of nanoparticles.

**Hydrogel Morphological Testing:** The freeze-dried hydrogel samples of appropriate sizes were sputter-coated with gold for 3 minutes at 20 mA, and their microstructures were observed using scanning electron microscopy (SEM) measurement methods.

**Rheological Tests:** Hydrogel samples were prepared using 3%PAMB-G-TK and 1.5% 4-arm-PEG-SG. The samples were detected by rotational rheometer (MARS 60, Haake, Germany), which mainly included storage modulus (G’) and loss modulus (G’’) of hydrogels in the Time sweeps (0-20 min), temperature (37°C), angular frequency range of (0.1-100 rad s^−1^) and frequency-shear rate sweeps (0-100 S^-1^).

**Hydrogel** **Degradation Test:** Different freeze-dried PAMB-G-TK/PEG-SG hydrogel samples were weighed to obtain W_0_, and then immersed in various PBS solutions containing different concentrations of H_2_O_2_ (0, 0.05, 0.1, and 0.5 mM) at 37°C. At predetermined time intervals, the samples were removed from the culture medium, freeze-dried, and weighed to obtain W_t_. Therefore, the weight remaining percentage of the hydrogel samples was calculated using the following equation.

Weight remaining (%) = (W_0_ − W_t_) / W_0_ × 100% (2)

**Hydrogel Liposome Release Assay:** Lipo-IL11 with Cy5.5 labeling was prepared to study the leakage of Lipo-IL11 from the hydrogels [4]. The Lipo-IL11 liposomes loaded in PAMB-G-TK/PEG-SG hydrogels were immersed in PBS containing different concentrations of H_2_O_2_, (0, 0.05, 0.1, 0.5 mM), respectively. At predetermined time intervals, 1 mL samples were taken from the solution and replaced with 1 mL of corresponding fresh solution. Finally, the absorbance of the solution containing Lipo-IL11 with Cy5.5 labelling was measured at a wavelength of 680 nm using a UV-vis spectrophotometer. The cumulative release curve of Lipo-IL11 in the hydrogel was obtained using a calibration curve of Lipo-IL11.

**Cell Culture**: A panel of OS cell lines and macrophage utilized in this study including 143B, K7M2, SJSA and U2OS, were purchased from American Type Culture Collection (ATCC, USA). L929, HUVECs, NIH3T3 and RAW264.7 cells were from the Cell Bank of the Chinese Academy of Sciences (Shanghai, China). Dendritic cells (DCs) were from Shanghai Cancer Center of Fudan University. All cells cultured in complete Dulbecco’s modified Eagle medium (DMEM) (10-013-CV, Corning, USA) medium at 37°C in a 5% CO_2_-humidified incubator.

***In Vitro* ROS Generation:** 143B cells were exposed to GSH (10 mM) and treated with PBS, Lipo-IL11, Mn@Lipo-IL11, Cis/Mn@Lipo-IL11, Cis/Mn@Lipo-IL11+NH, and Cis/Mn/NH@PGP for 24 hours at 37°C in the dark. Afterward, they were stained with DCFH-DA (10 μM) for 30 minutes, and fluorescence signals were captured to determine the intracellular ROS generation by using ImageJ software.

**Cell Apoptosis Detection:** Apoptosis of 143B cancer cells under different treatment conditions was stained using the Annexin V-FITC/PI assay kit. The cells were analyzed by single or multicolor staining using the BD LSRFortessa flow cytometer (BD, USA). Further analysis and plotting of the raw data files were performed using FlowJo v10.8 software (BD, USA).

**Cell Migration and** **Invasion Assays:** Cell migration and invasion capabilities were assessed using scratch wound healing assays or transwell invasion assays. For the scratch wound healing assay, 143B cells were seeded in a 6-well plate. When the cells reached approximately 80% confluence, a scratch was made using a sterile 200 µL pipette tip. After treatment with different materials, the cells were washed three times with PBS and observed under inverted fluorescence microscopy at 0 hour and 24 hours to assess cell migration.

For the transwell invasion assay, 143B cells were seeded into the upper chamber (8 μm) of a 24-well transwell insert pre-coated with Matrigel (#354234, Corning, USA) and incubated overnight at 37°C. The cells were cultured with serum-free culture medium in the upper chamber, while complete culture medium containing different materials was added to the lower chamber of the 24-well plate. After 24 hours, the cells in the upper chamber were removed using a cotton swab. The transwell chambers were then fixed with methanol for 10 minutes and stained with crystal violet for 20 minutes. After several washes with PBS buffer, images were captured under inverted fluorescence microscopy (Carl Zeiss, Germany). The invaded cells were further counted and compared using Image J software**.**

**Transcriptome Analysis by RNA Sequencing (RNA-seq):** Total RNA was extracted from 143B cells using TRIzol. Subsequently, purified RNA was reverse transcribed to generate complementary DNA (cDNA) using the RNeasy Micro Kit (QIAGEN, Valencia, CA). Each experiment was replicated three times. RNA-seq was performed using fastb software Technology (Shanghai, China).

**Evaluation of JAK2 and STAT3 Expression *In Vitro*:** 143B cells were seeded in a 12-well plate and treated with PBS, Lipo-IL11, Mn@Lipo-IL11, Cis/Mn@Lipo-IL11, Cis/Mn@Lipo-IL11+NH, and Cis/Mn/NH@PGP for 24 hours. Subsequently, cell lysates were prepared using RIPA lysis buffer to extract proteins, and their concentrations were determined using the BCA Protein Assay Kit. Equal amounts of protein were separated by gel electrophoresis, followed by protein-blots analysis to evaluate the expression of JAK2 and STAT3 proteins.

**Immunofluorescence Analysis of Macrophage Polarization:** RAW264.7 cells were cultured in a 24-well plate and subjected to the aforementioned material treatments. Following treatment, the cells were fixed with 4% paraformaldehyde (PFA) for 30 minutes, then washed three times with PBS. Permeabilization was performed using 0.1% Triton X-100, followed by washing with PBS. Subsequently, the cells were blocked with 5% BSA at room temperature for 1 hour and then incubated with primary antibodies of CD86 and CD206 at 4°C for overnight. Then, the cells were treated with secondary antibodies for 1 hour at room temperature, and observed using CLSM.

**Evaluation of Arg1, iNOS, and CD206 expression *In Vitro*:** RAW264.7 cells were treated under different conditions, followed by lysis using ice-cold RIPA buffer containing protease and phosphatase inhibitors. Protein concentration was determined using the BCA Protein Assay Kit. Protein samples were loaded into SDS-PAGE wells and detected using the Bio-Rad electrophoresis system.

**RNA Extraction and Real-Time PCR Analysis:** After the treatment with different materials for 24 hours, total RNA was extracted from RAW264.7 using TRIzol. Subsequently, RNA reverse transcription and quantitative real-time PCR reactions (qPCR) were performed using the PrimeScript RT Reagent Kit and RT-PCR Kit (Takara, Japan). The relative mRNA expression levels were calculated using the comparative Ct method, with GAPDH serving as the internal reference. The primers for the target genes were designed and synthesized by Sangon Biotech (Shanghai) Co., Ltd. (China), detailed gene-specific primers were listed in Table S1.

**Evaluation of CRT, HMGB1, and MYC Expression *In Vitro*:** K7M2 cells were treated under different conditions, followed by lysis using ice-cold RIPA buffer containing protease and phosphatase inhibitors. Protein concentration was determined using the BCA Protein Assay Kit. Protein samples were loaded into SDS-PAGE wells and detected using the Bio-Rad electrophoresis system.

**Animal Care:** SD rats, Balb/c nude mice and mice (5-6 weeks) were obtained from Shanghai Jihui Biotechnology Co., Ltd. (Shanghai, China). The animal studies were conducted following the guidelines of the Institutional Animal Care and Use Committee and approved by the Ethics Committee of the First People’s Hospital, Shanghai Jiao Tong University School of Medicine (2023SQ084).

***In Vivo* Detection of Tumor ROS Production:** In the 143B OS mouse model, injections of PBS, PGP, Mn@PGP, Cis/Mn/NH@PGP, Mn/NH@PGP, Cis/Mn/NH@PGP (25 mg/kg) were administered. DCFH-DA (25 μL, 5 μM) was injected 24 hours post-treatment to detect ROS in the tumor (n = 5). Immediately after treatment, mice were euthanized, and tumors were collected for frozen sectioning. Fluorescence images of tumor sections were then captured using CLSM.

**Biodegradation of Cis/Mn/NH@PGP hydrogel *In Vivo*:** 100 mg Cis/Mn/NH@PGP hydrogel were injected locally adjacent to SD rats, respectively (n = 3). The hydrogel was injected under the skin of the mice and the degradation of the hydrogel was observed in the mice for 0, 1, 3 and 7 days and weighed its remaining mass.

**Biological Safety Assessment:** To evaluate the biosafety of the nanomaterials, we monitored the body weight of the mouse models continuously for 24 days (n = 5). On the 24th day post-treatment, the mice were euthanized, and liver, kidney, spleen, and heart tissues were collected for H&E staining to observe their histological morphology for safety assessment. Blood samples were collected from mice after different treatments for analysis of blood routine, blood biochemistry, and other functional indicators.

**Biosafety Assessment:** To evaluate the biosafety of nanocomposite hydrogel, body weights of mouse models were recorded for 24 days (n = 5). On day 24 after treatments, mice were euthanized to collect kidney, spleen and heart for H&E staining to observe the histological morphologies for safety evaluation. After different treatments, the blood samples were collected from mice, and analysis of blood routine, blood biochemistry, and liver and kidney function indexes were conducted.

**S2. Supplementary Figures**


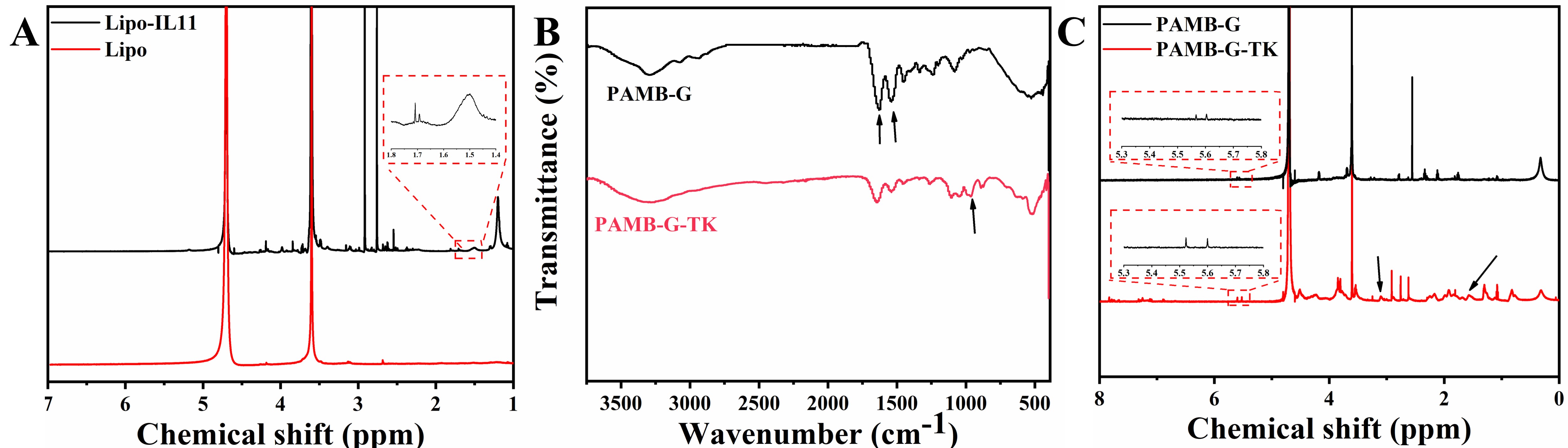


**Fig. S1** (A) ^1^H NMR spectra of Lipo and Lipo-IL11. (B) FTIR spectra of PAMB-G and PAMB-G-TK. (C) ^1^H NMR spectra of PAMB-G and PAMB-G-TK. The specific peak at 1.72 ppm in the ^1^H NMR spectrum confirmed the functionalization of IL11 peptide in Lipo-IL11 [5]. The FTIR spectrum of PAMB-G copolymer in Fig. S1B includes two characteristic peaks at 1644 and 1533 cm^−1^, corresponding to the amide I (C=O stretching vibration) and the amide II (N–H bending vibration) of gelatin, respectively [6]. In addition, the appearance of characteristic peaks at 887 cm^-1^ were corresponded to the C-S in TK, confirmed that the successful synthesis of PAMB-G-TK. The ^1^H NMR spectrum of PAMB-G copolymer exhibited signals in the range of 5.5–5.8 ppm, which were associated with the protonated N–H groups on PAMB. In the ^1^H NMR spectrum of PAMB-G-TK, the peaks at 3.1 ppm and 1.6 ppm are attributed to the presence of methyl groups (-CH_3_) in the TK chemical bond [4].


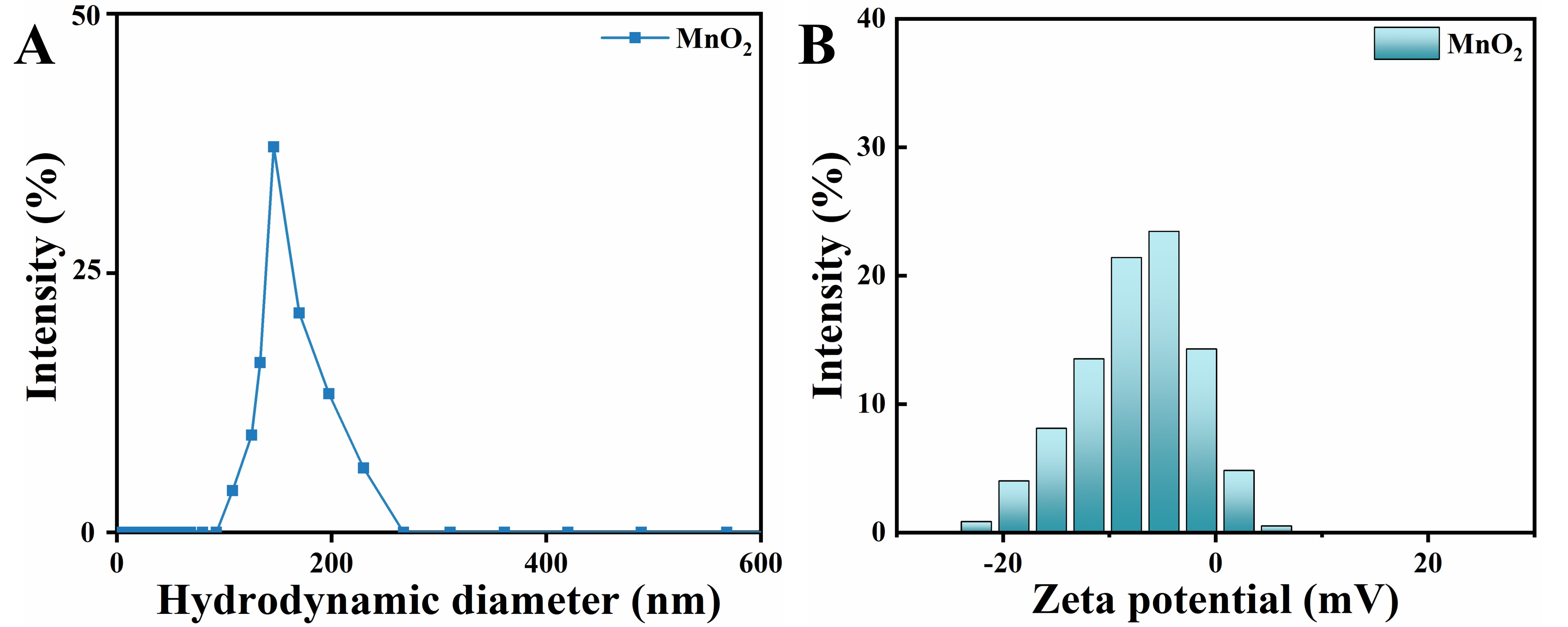


**Fig. S2** (A) The hydrodynamic diameter and (B) zeta potentials of MnO_2_ nanoparticles.





**Fig. S3** Zeta potentials of Lipo-IL11, Mn@Lipo-IL11, and Cis/Mn@Lipo-IL11 measured in aqueous solution.





**Fig. S4** The cisplatin release profile of Cis/Mn@Lipo-IL11 with or without GSH.


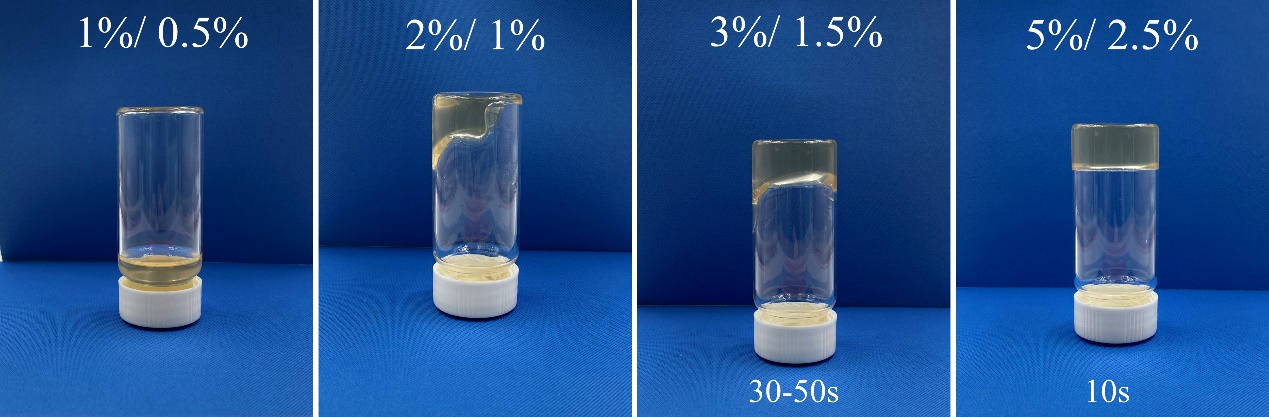


**Fig. S5** The gelation state images of hydrogels at different ratios of PAMB-G-TK and 4-arm-PEG-SG.


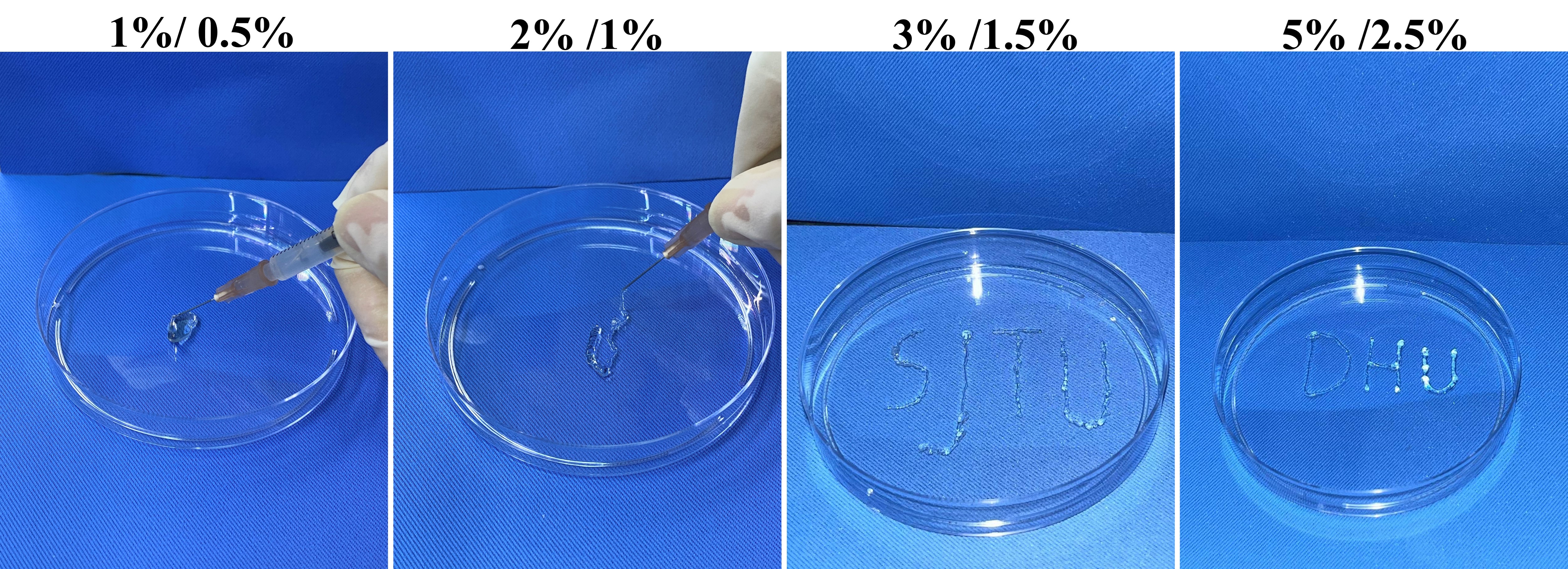


**Fig. S6** Photos of injectability and plasticity of hydrogels at different ratios of PAMB-G-TK and 4-arm-PEG-SG using 25G needle.


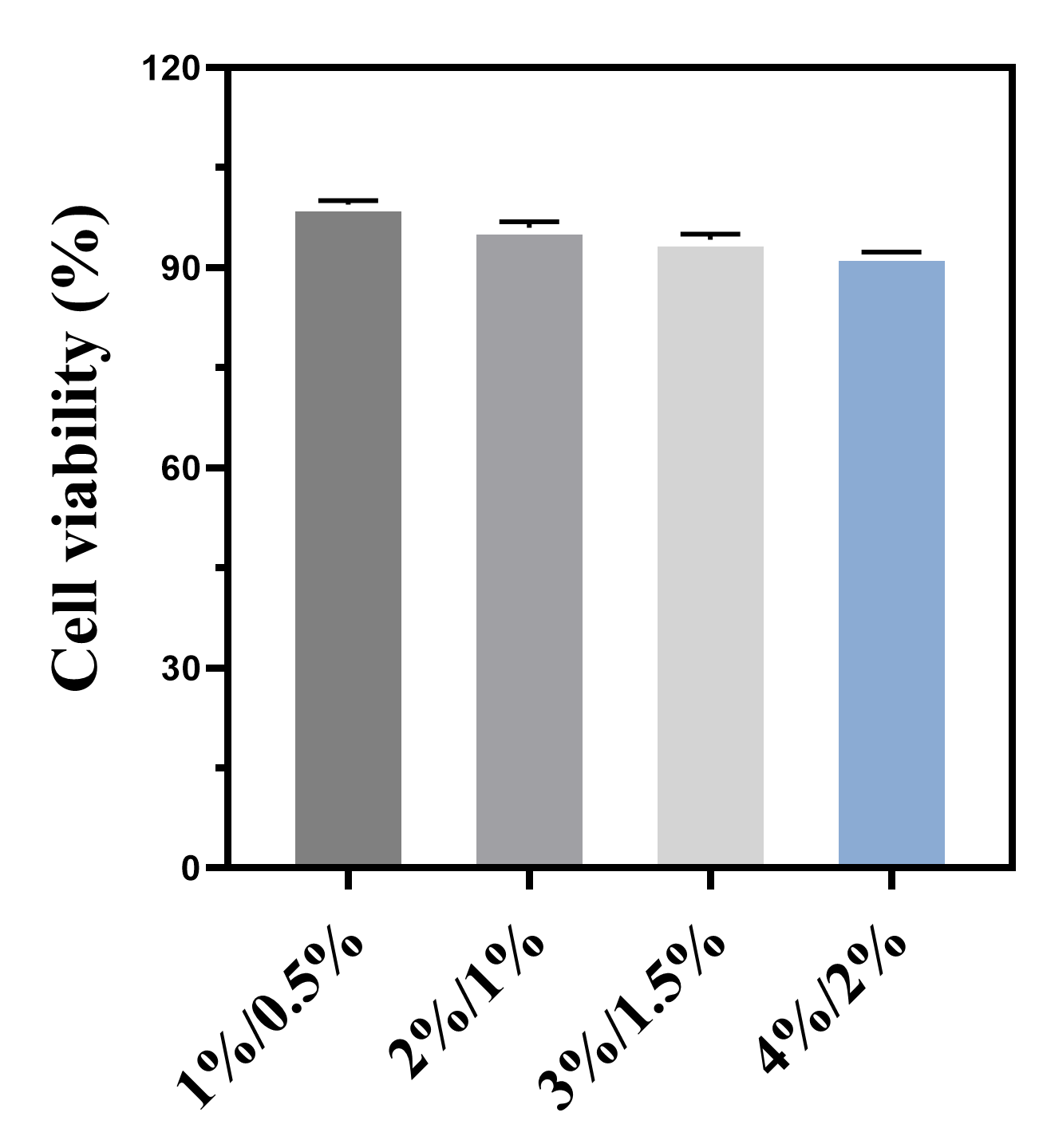


**Fig. S7** Cell viability of L929 cells treated with different ratios of PAMB-G-TK and 4-arm-PEG-SG.





**Fig. S8** The Lipo release behavior of Lipo-IL11 from Cis/Mn@PGP hydrogel at different concentrations of H_2_O_2_.


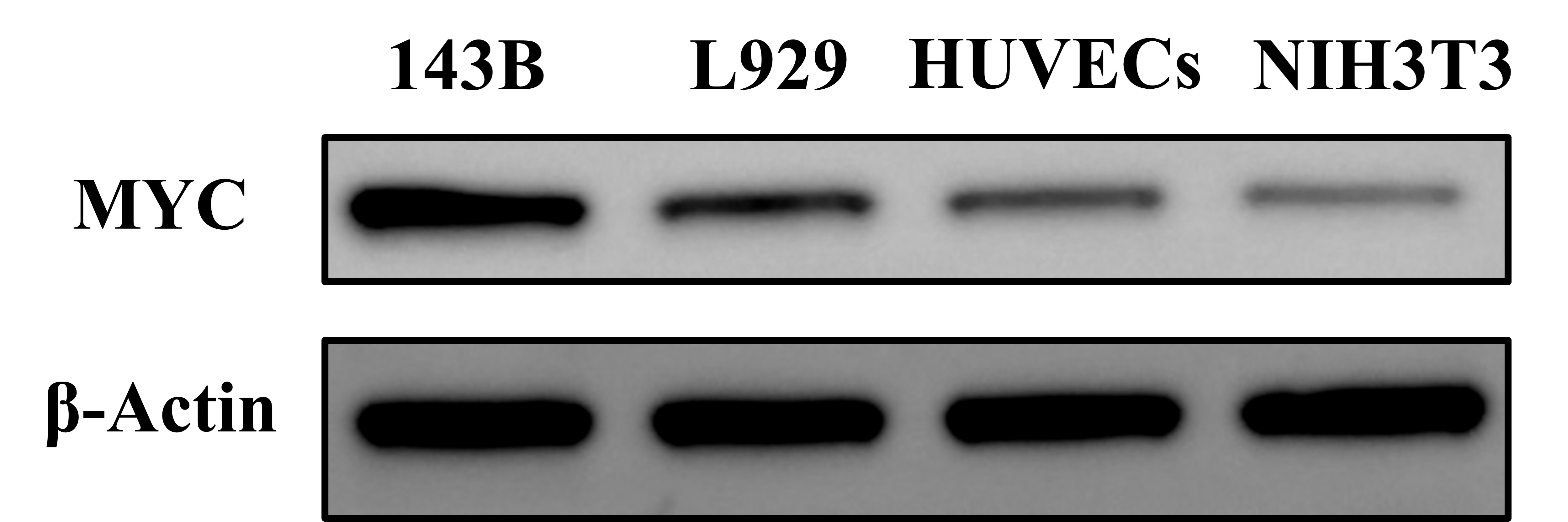


**Fig. S9** The expression levels of MYC protein in tumor cells (143B) and normal cells (L929, HUVECs and NIH3T3).


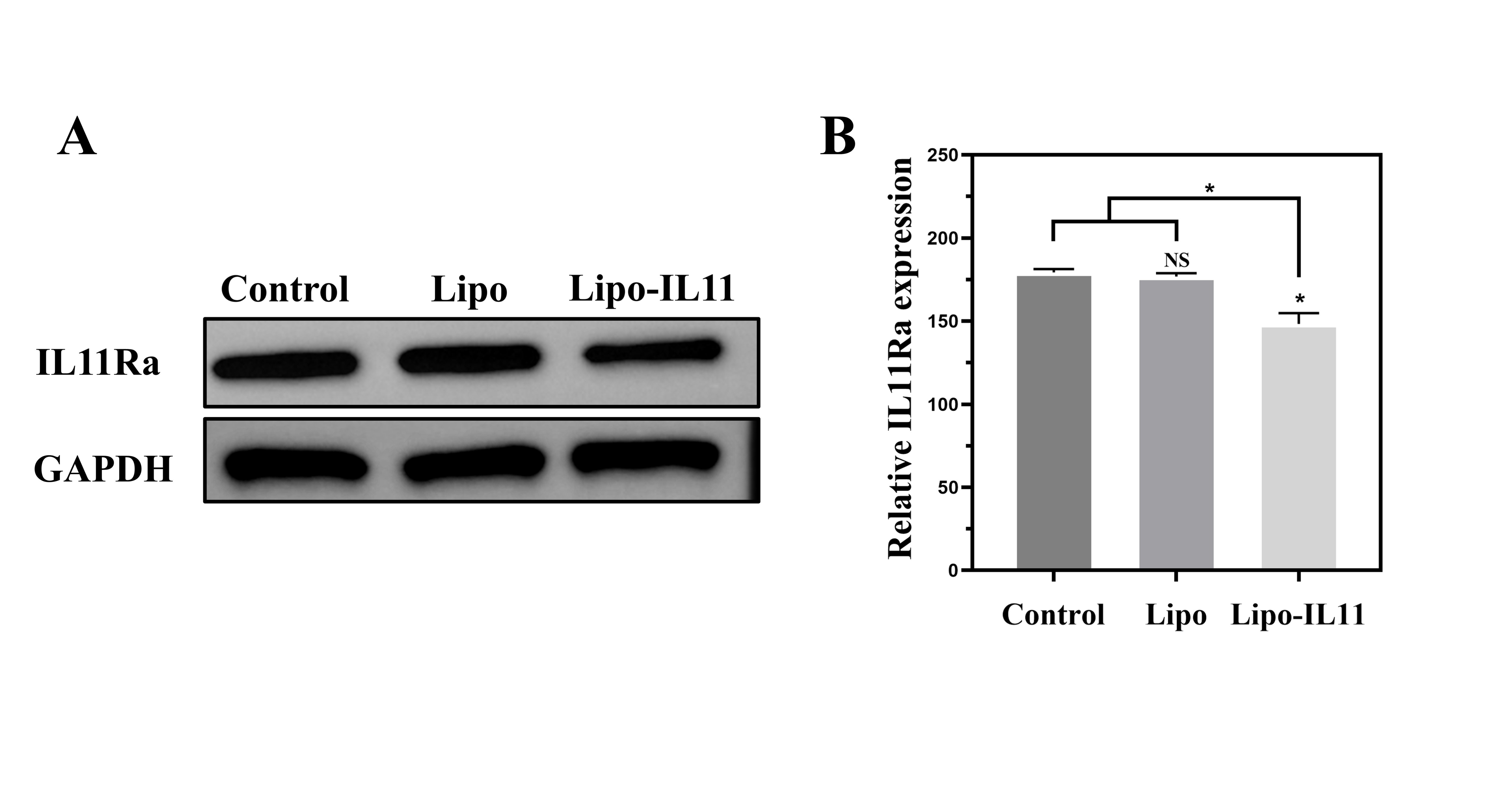


**Fig. S10** The expression level of IL11Ra protein in 143B cells after treatment with different materials. ns: no significance, *P < 0.05, compared with Control group.


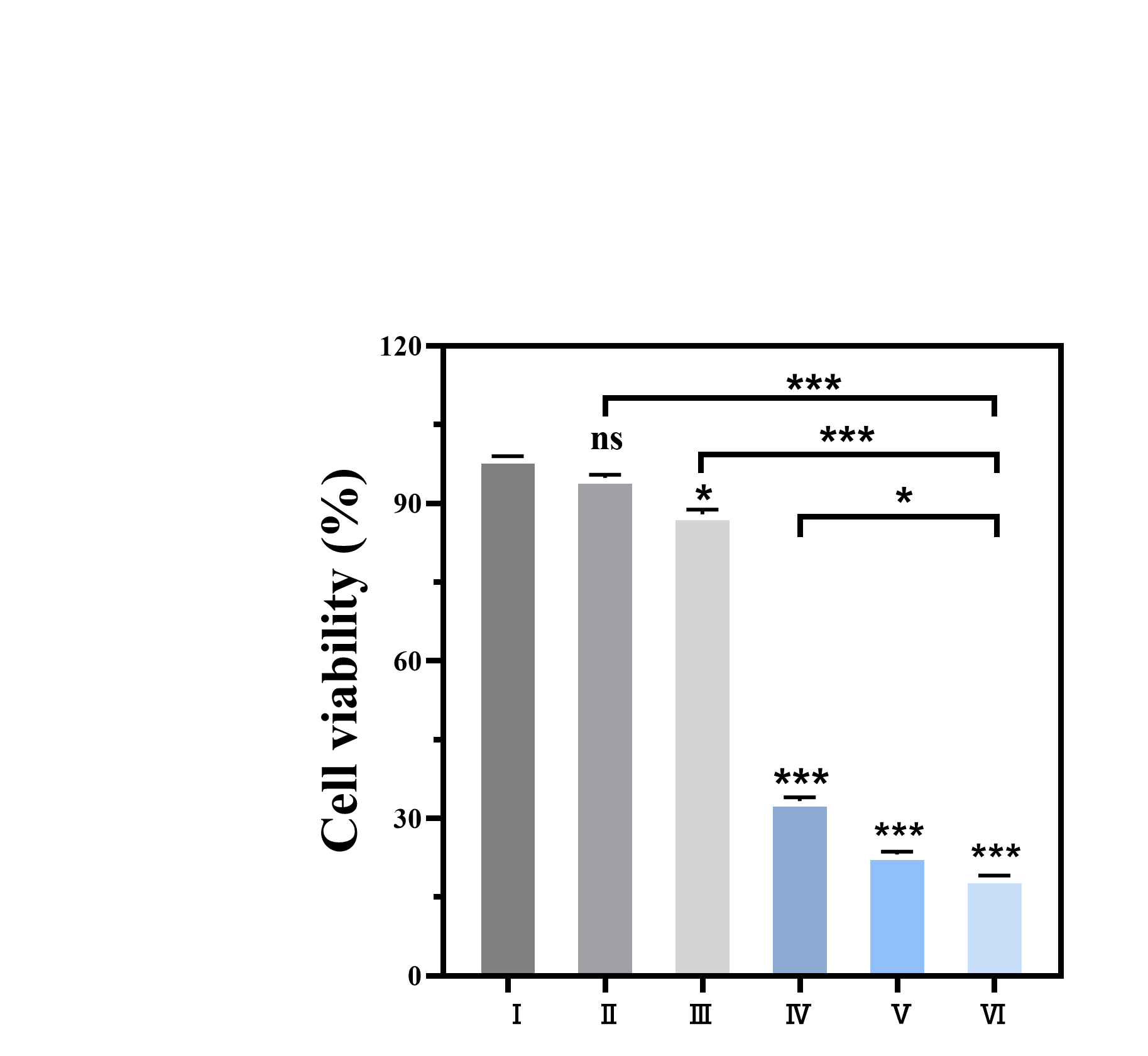


**Fig. S11** Cell viability of 143B cells after treatment with different materials, Ⅰ: Control, Ⅱ: Lipo-IL11, Ⅲ: Mn@Lipo-IL11, Ⅳ: Cis/Mn@Lipo-IL11, Ⅴ: Cis/Mn@Lipo-IL11+NH, Ⅵ: Cis/Mn/NH@PGP. ns: no significance, *P < 0.05, **P < 0.01, ***P < 0.001, compared with Control group.


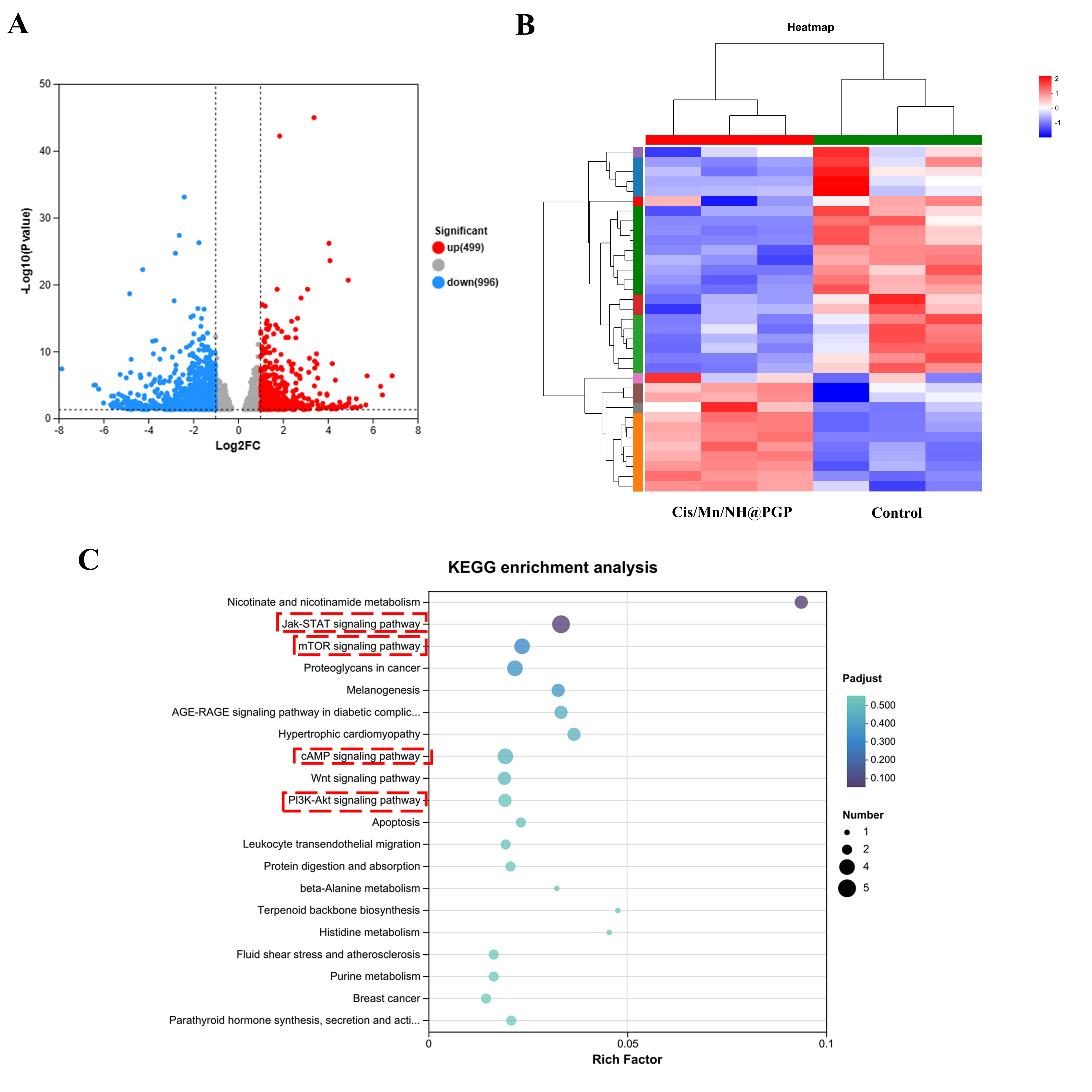


**Fig. S12** (A) Volcano plot showing the upregulated/downregulated genes after treatment with Cis/Mn/NH@PGP compared to the Control group. (B) Cluster heatmap displaying the treatment effect of Cis/Mn/NH@PGP compared to the Control group. (C) KEGG analysis of downregulated genes after treatment with Cis/Mn/NH@PGP compared to the Control group.


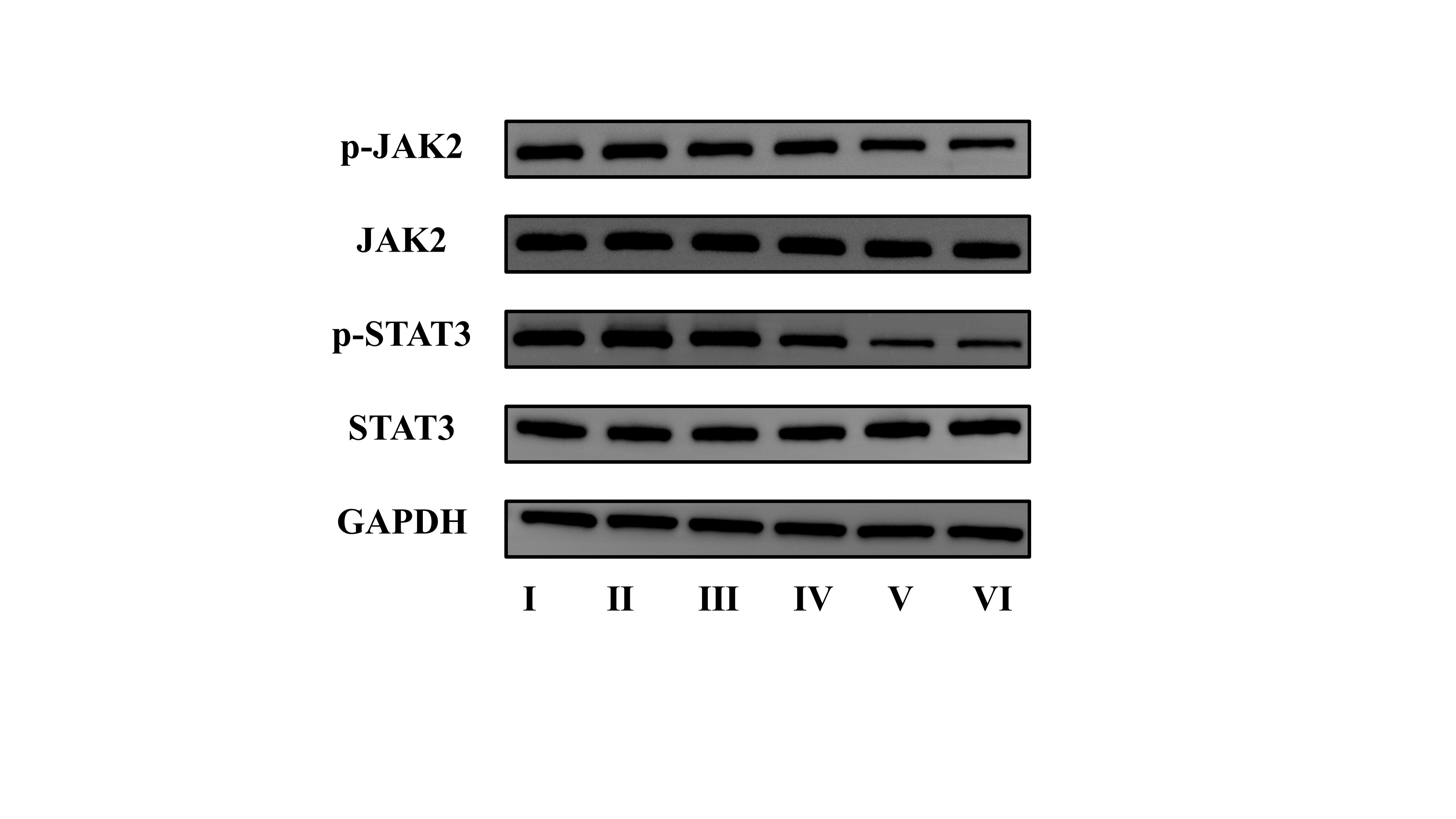


**Fig. S13** Western Blot analysis of JAK-STAT3 signaling pathway expression levels in 143B cells after treatment with different materials. Ⅰ: Control, Ⅱ: Lipo-IL11, Ⅲ: Mn@Lipo-IL11, Ⅳ: Cis/Mn@Lipo-IL11, Ⅴ: Cis/Mn@Lipo-IL11+NH, Ⅵ: Cis/Mn/NH@PGP.


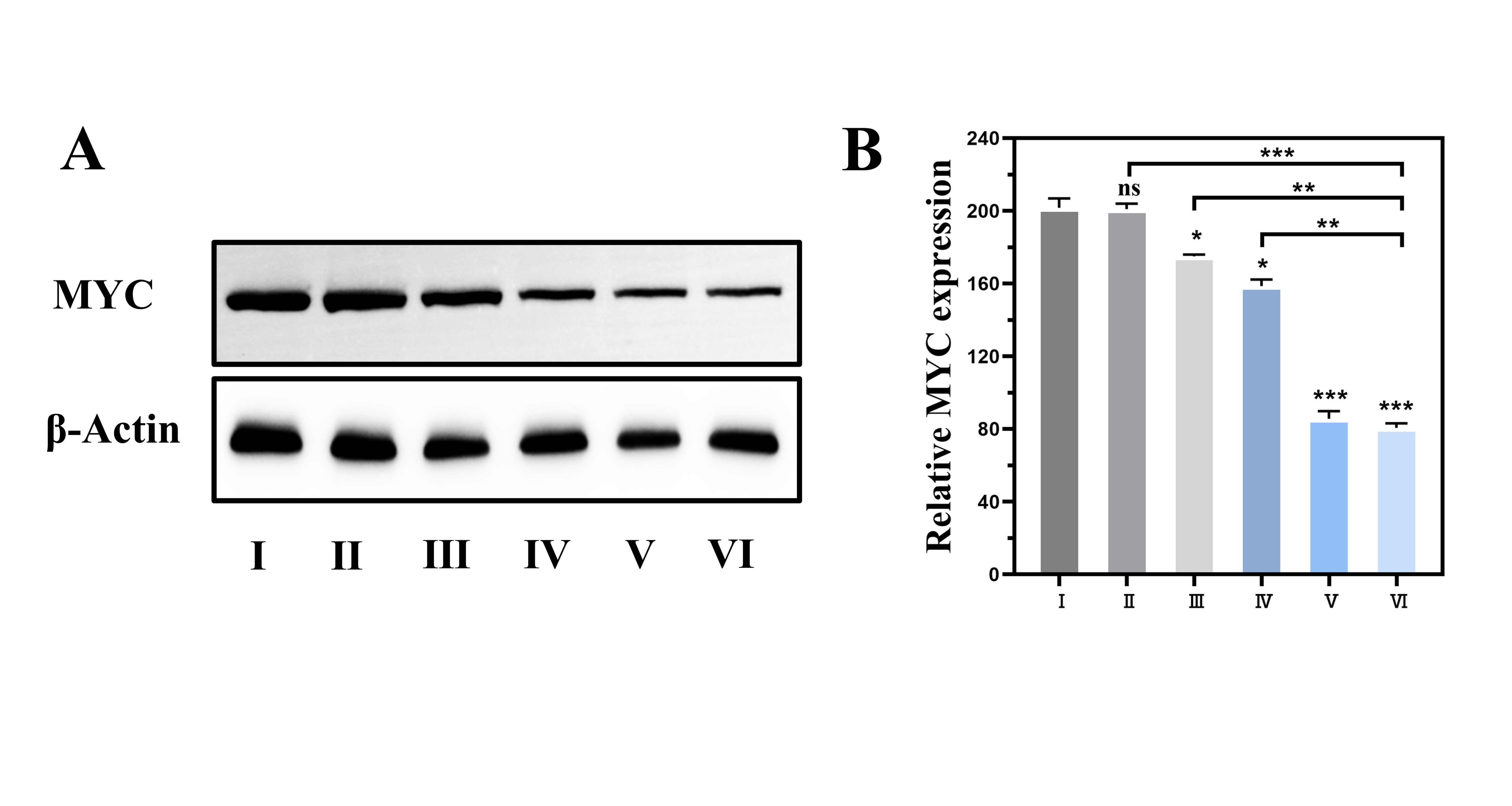


**Fig. S14** The expression level of MYC protein in 143B cells after treatment with different materials. (A) Western Blot image. (B) Quantitative analysis of relative expression. Ⅰ: Control, Ⅱ: Lipo-IL11, Ⅲ: Mn@Lipo-IL11, Ⅳ: Cis/Mn@Lipo-IL11, Ⅴ: Cis/Mn@Lipo-IL11+NH, Ⅵ: Cis/Mn/NH@PGP. ns: no significance, *P < 0.05, **P < 0.01, ***P < 0.001, compared with Control group.


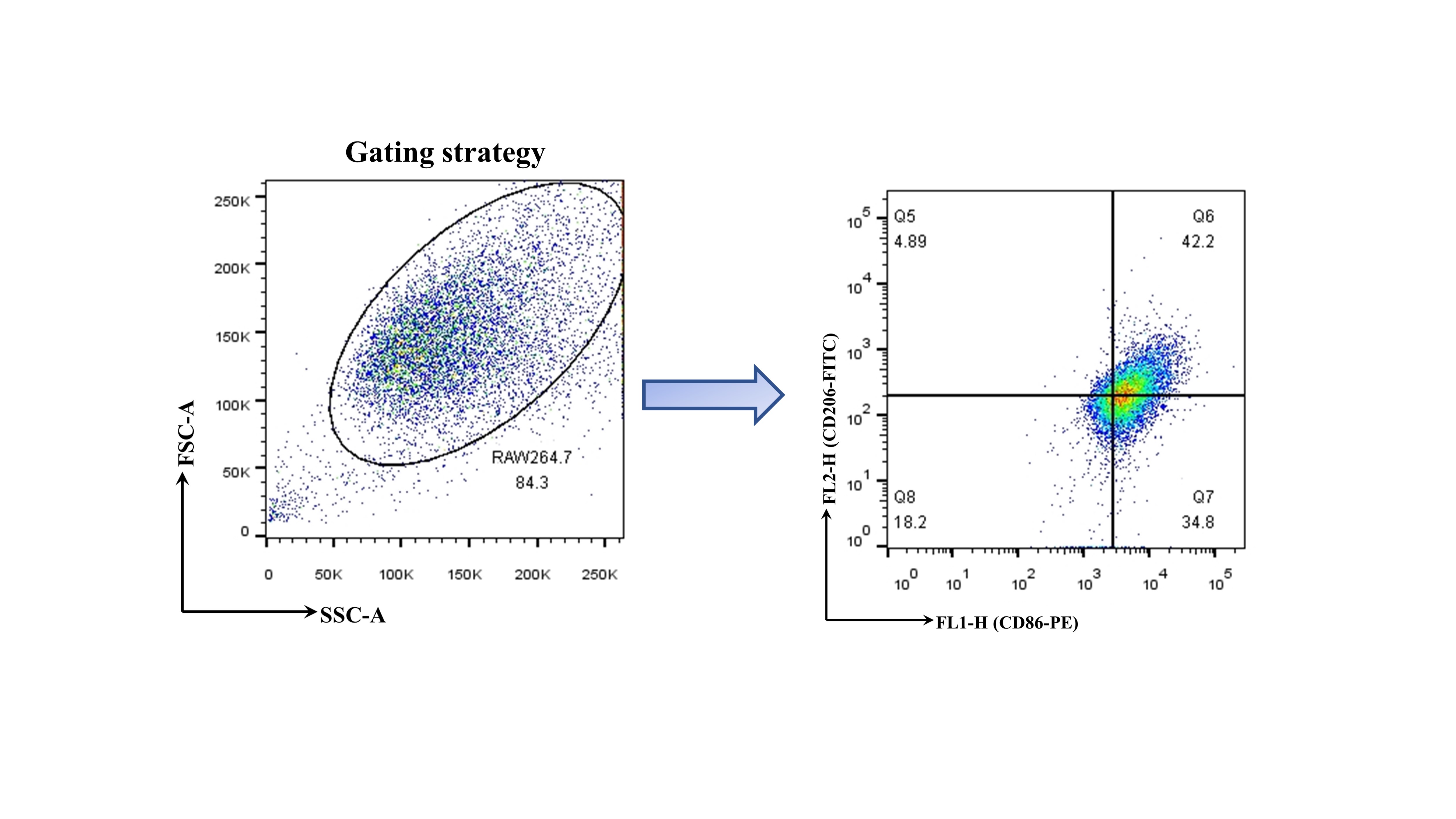


**Fig. S15** Gating strategy of flow cytometry analysis for RAW264.7 polarization (M1-type macrophages CD86^+^ and M2-type macrophages CD206^+^).


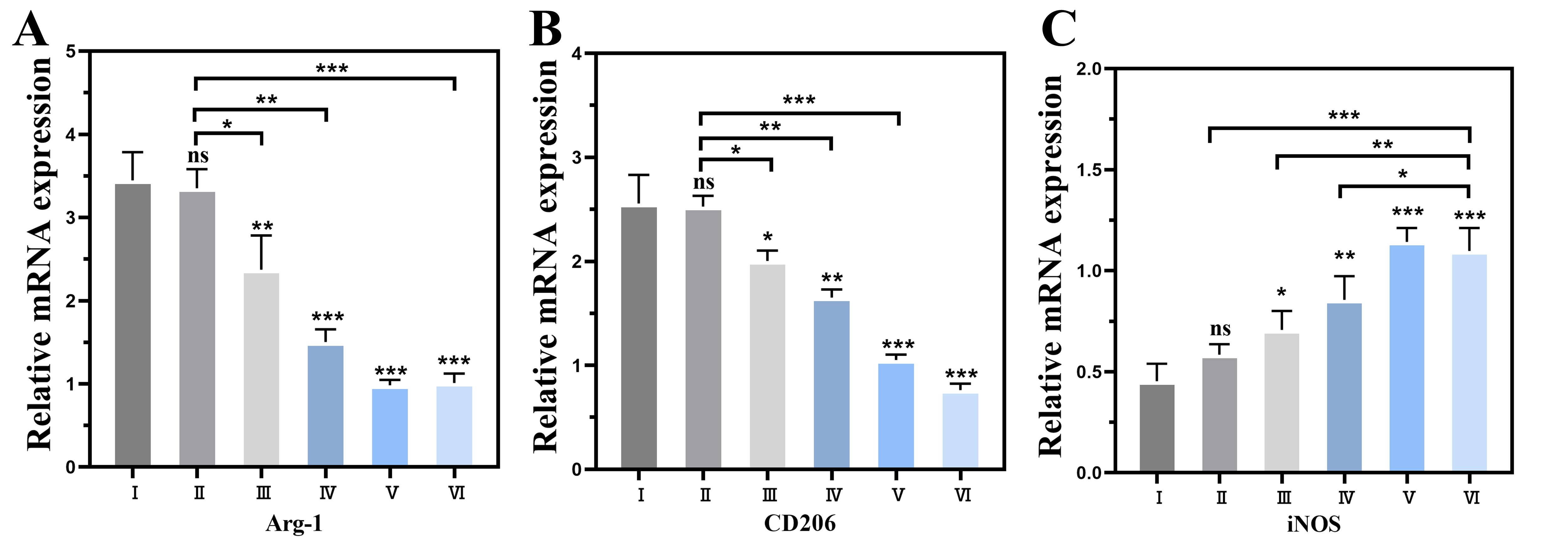


**Fig. S16** RAW264.7 polarization after treatment with different materials by RT-qPCR analysis: (A) Arg-1, (B) CD206, (C) iNOS. Ⅰ: Control, Ⅱ: Lipo-IL11, Ⅲ: Mn@Lipo-IL11, Ⅳ: Cis/Mn@Lipo-IL11, Ⅴ: Cis/Mn@Lipo-IL11+NH, Ⅵ: Cis/Mn/NH@PGP. ns: no significance, *P < 0.05, **P < 0.01, ***P < 0.001, compared with Control group.


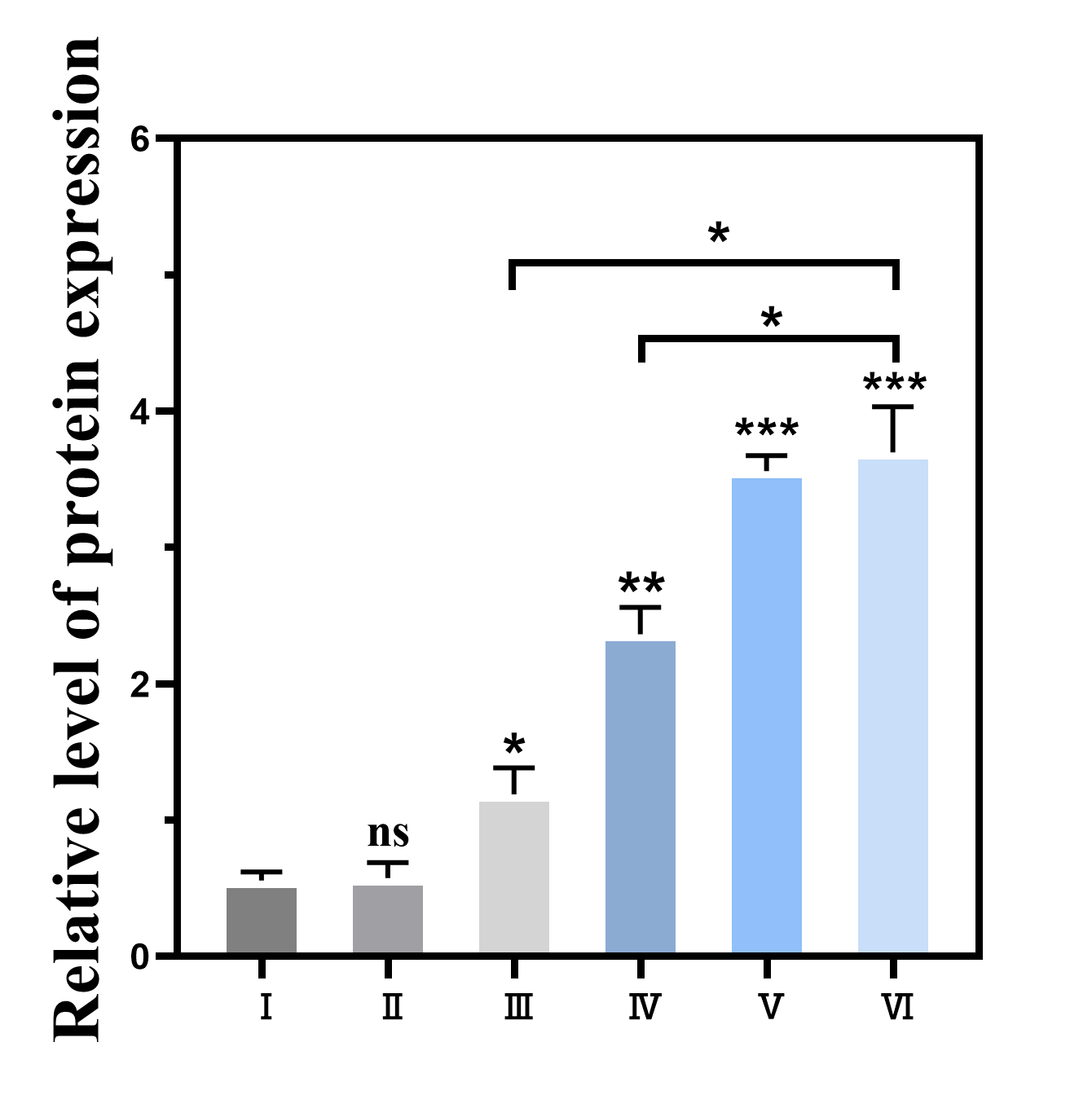


**Fig. S17** ELISA analysis of HMGB1 expression levels in K7M2 cell culture medium. Ⅰ: Control, Ⅱ: Lipo-IL11, Ⅲ: Mn@Lipo-IL11, Ⅳ: Cis/Mn@Lipo-IL11, Ⅴ: Cis/Mn@Lipo-IL11+NH, Ⅵ: Cis/Mn/NH@PGP. ns: no significance, *P < 0.05, **P < 0.01, ***P < 0.001, compared with Control group.


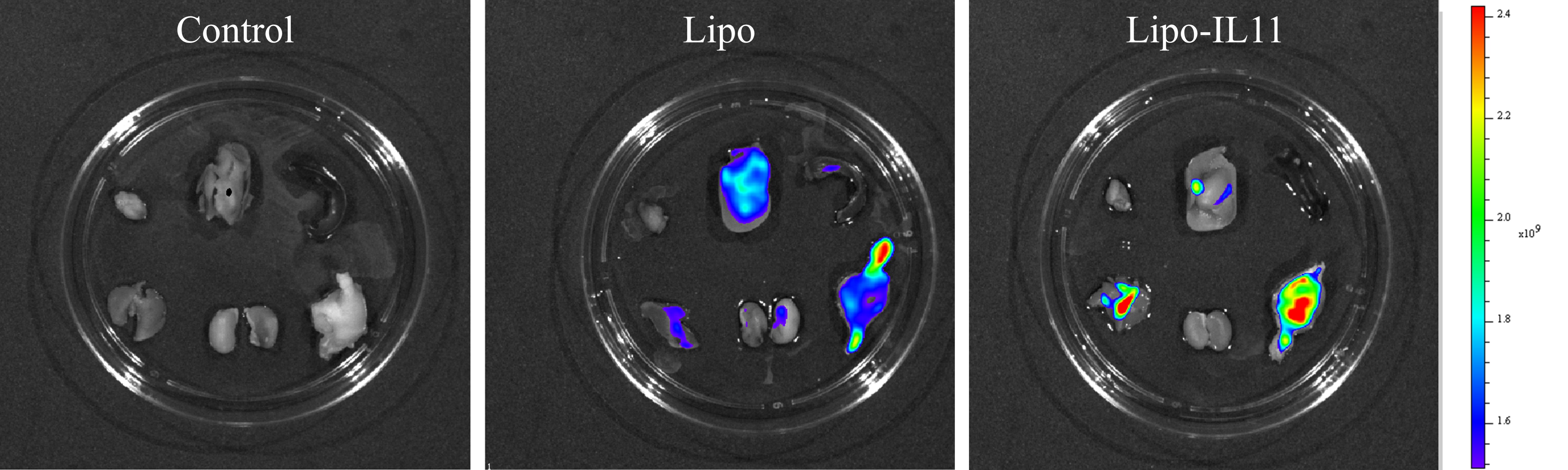


**Fig. S18** Fluorescence images of major organs and tumors in mice after treatment with Lipo and Lipo-IL11 (labelled with Cy5.5) for 24 hours.


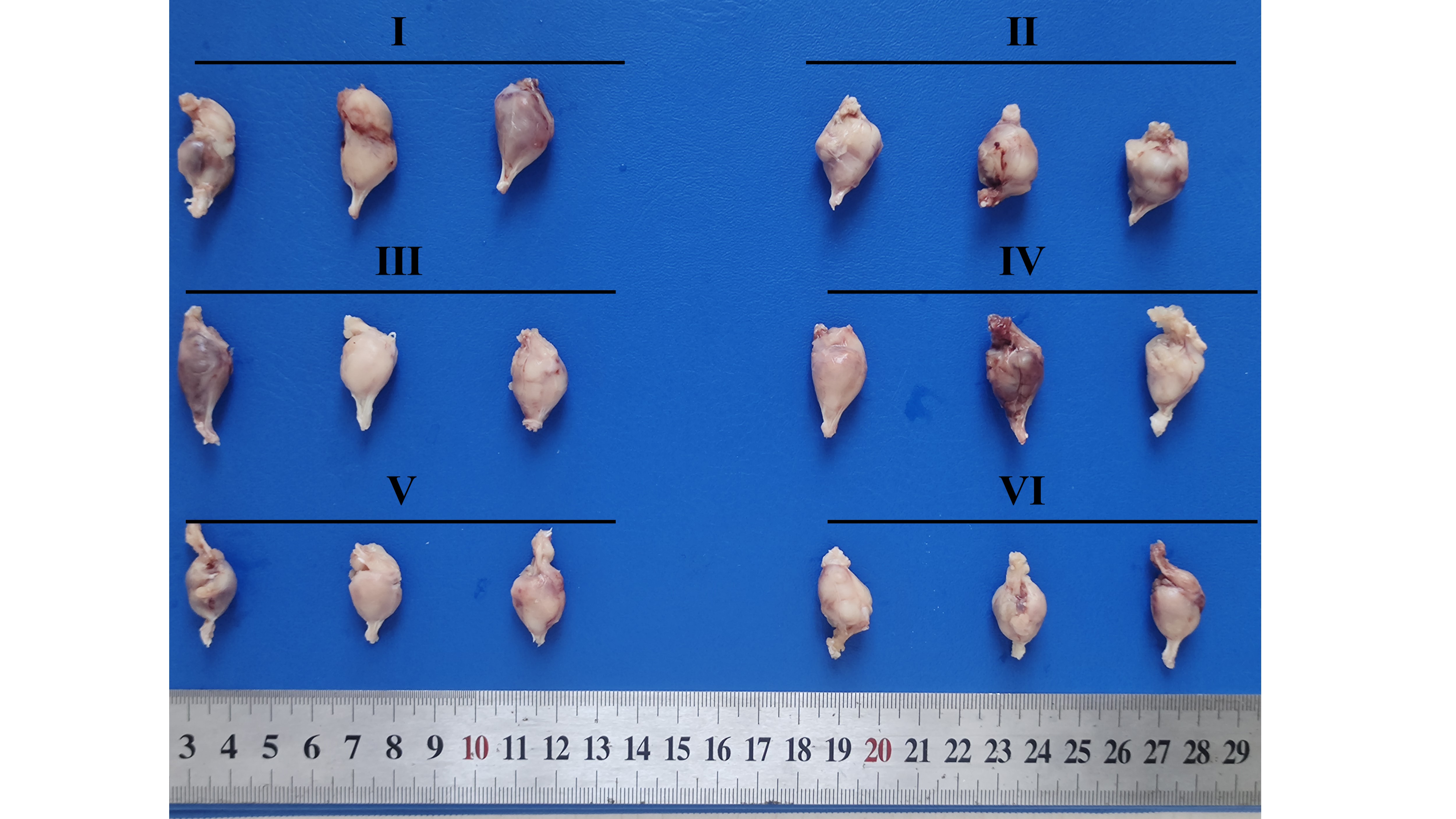


**Fig. S19** Representative images of tumor tissue in 143B-bearing OS model after treatment with different materials for 24 days. Ⅰ: Control, Ⅱ: PGP, Ⅲ: Mn@PGP, Ⅳ: Cis/Mn@PGP, Ⅴ: Mn/NH@PGP, Ⅵ: Cis/Mn/NH@PGP.


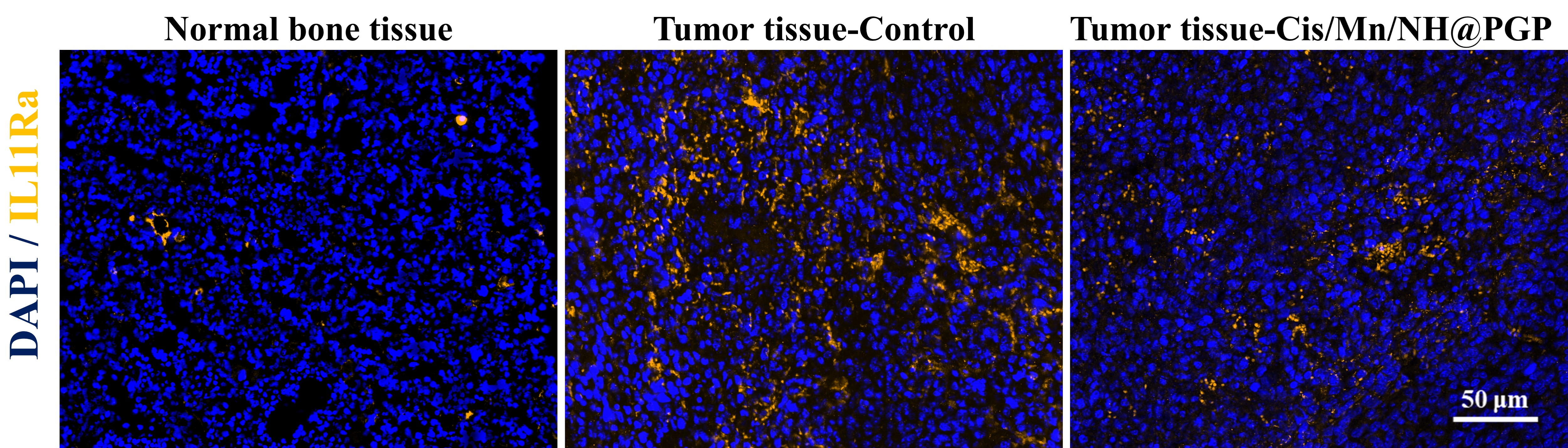


**Fig. S20** Representative images of tumor tissue in 143B-bearing OS model and normal bone tissue after treatment with different materials for 24 days.


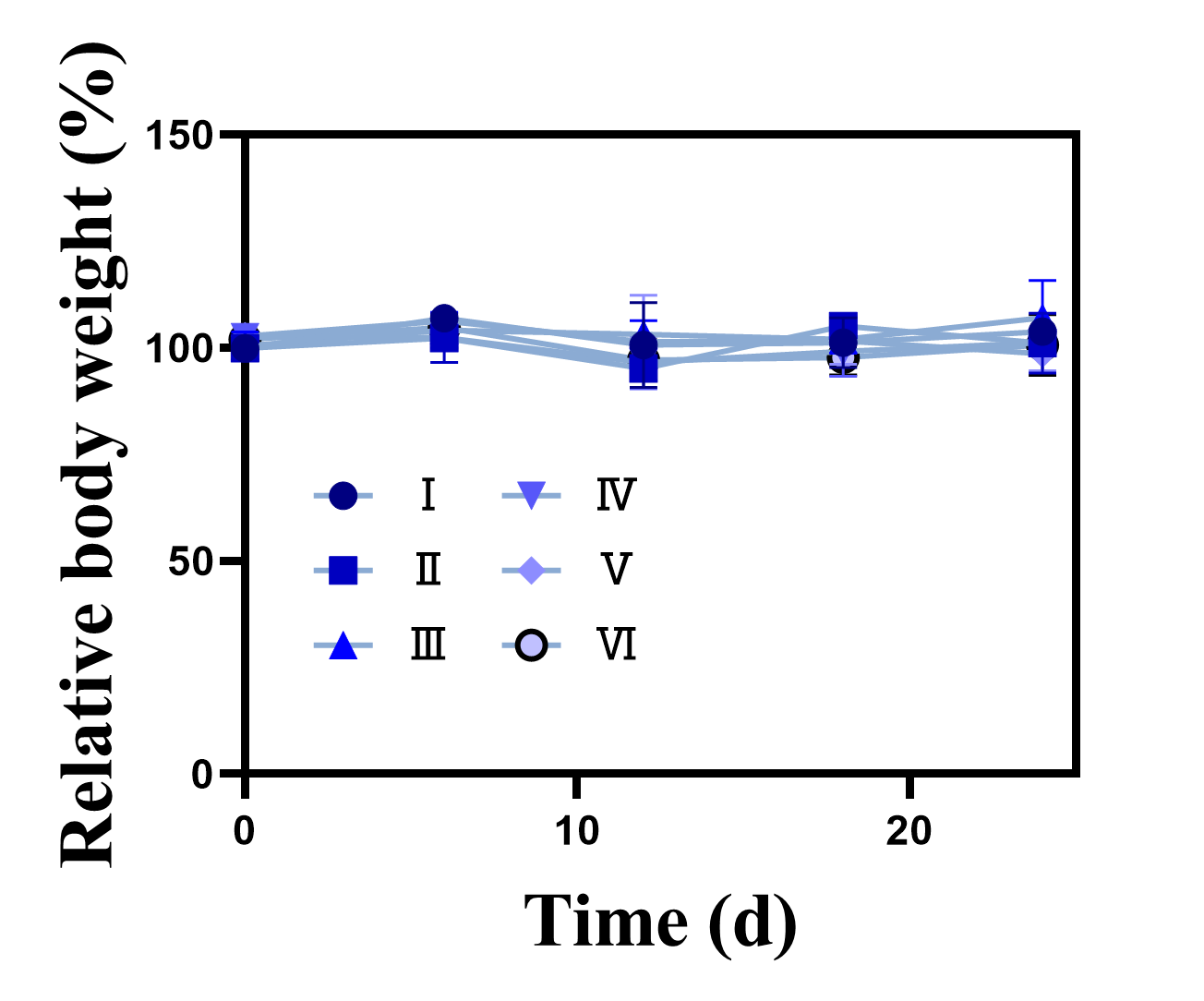


**Fig. S21** The weight changes of nude mice in 143B-bearing OS model after treatment with different materials. Ⅰ: Control, Ⅱ: PGP, Ⅲ: Mn@PGP, Ⅳ: Cis/Mn@PGP, Ⅴ: Mn/NH@PGP, Ⅵ: Cis/Mn/NH@PGP.


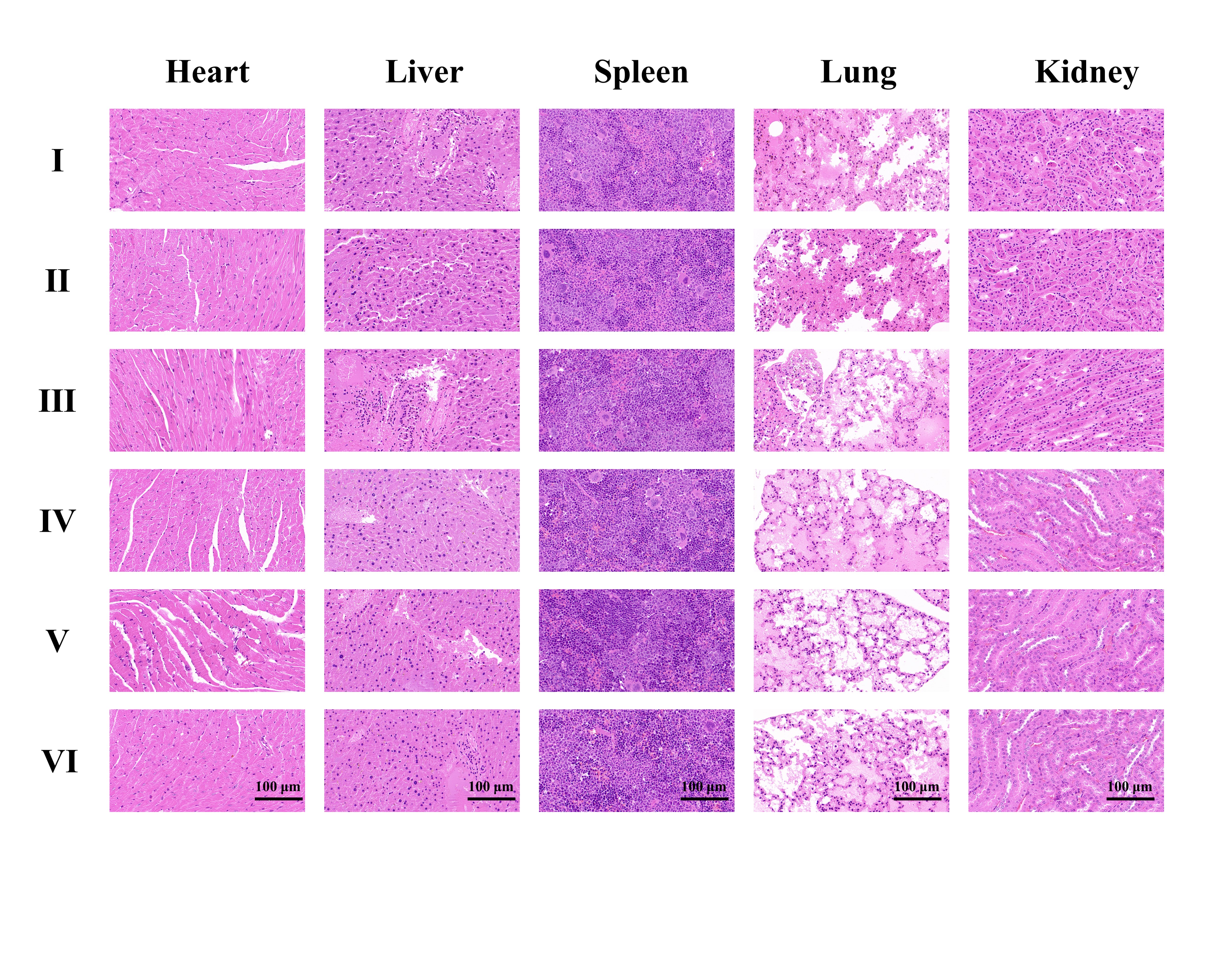


**Fig. S22** Representative images of major organs (heart, liver, spleen, lung and kidney) in nude mice after treatment with different materials for 24 days. Ⅰ: Control, Ⅱ: PGP, Ⅲ: Mn@PGP, Ⅳ: Cis/Mn@PGP, Ⅴ: Mn/NH@PGP, Ⅵ: Cis/Mn/NH@PGP.


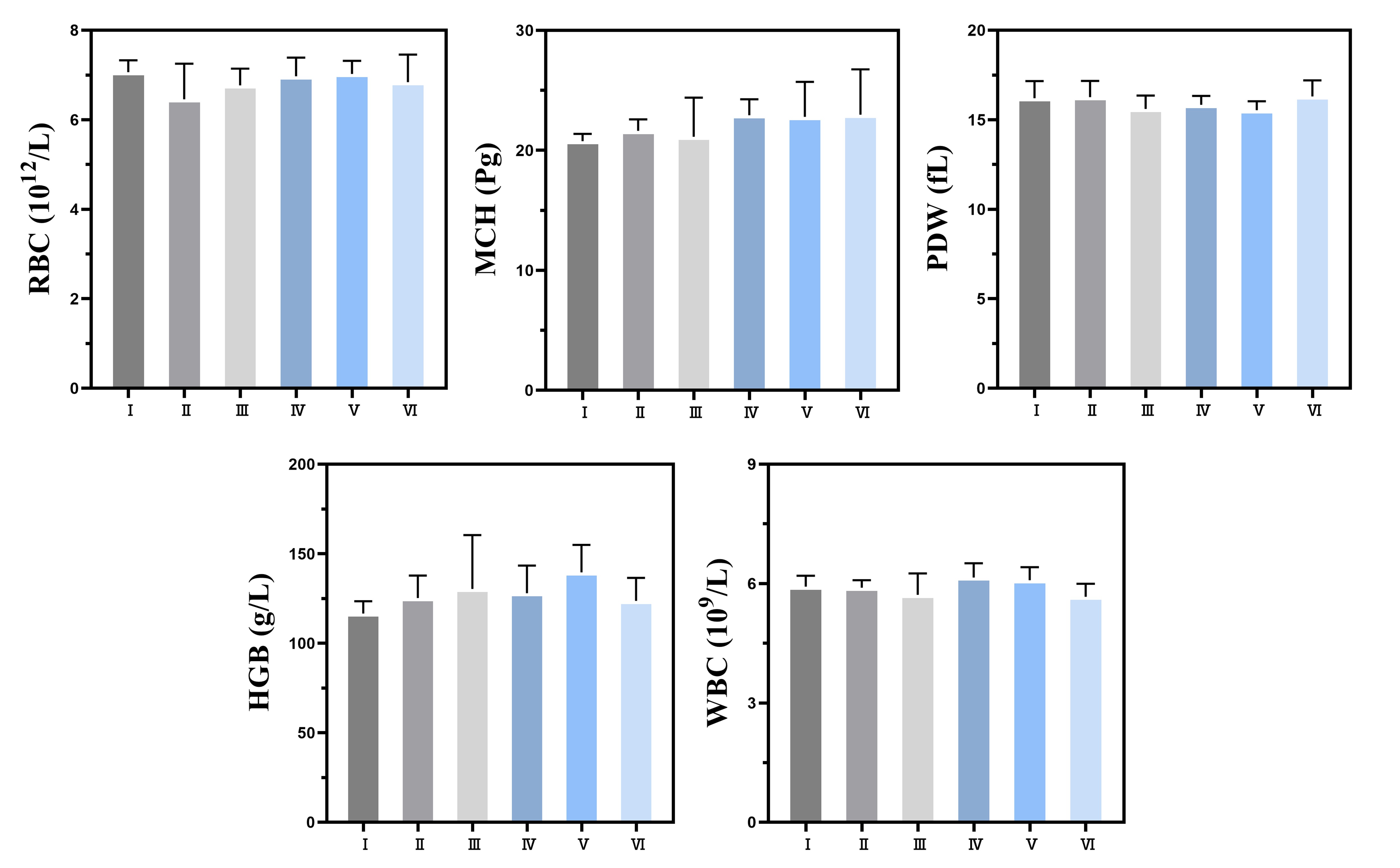


**Fig. S23** Blood routine examination of nude mice after treatment with different materials for 14 days, including red blood cell count (RBC), mean corpuscular hemoglobin (MCH), platelet distribution width (PDW), hemoglobin concentration (HGB), and white blood cell count (WBC). Ⅰ: Control, Ⅱ: PGP, Ⅲ: Mn@PGP, Ⅳ: Cis/Mn@PGP, Ⅴ: Mn/NH@PGP, Ⅵ: Cis/Mn/NH@PGP.


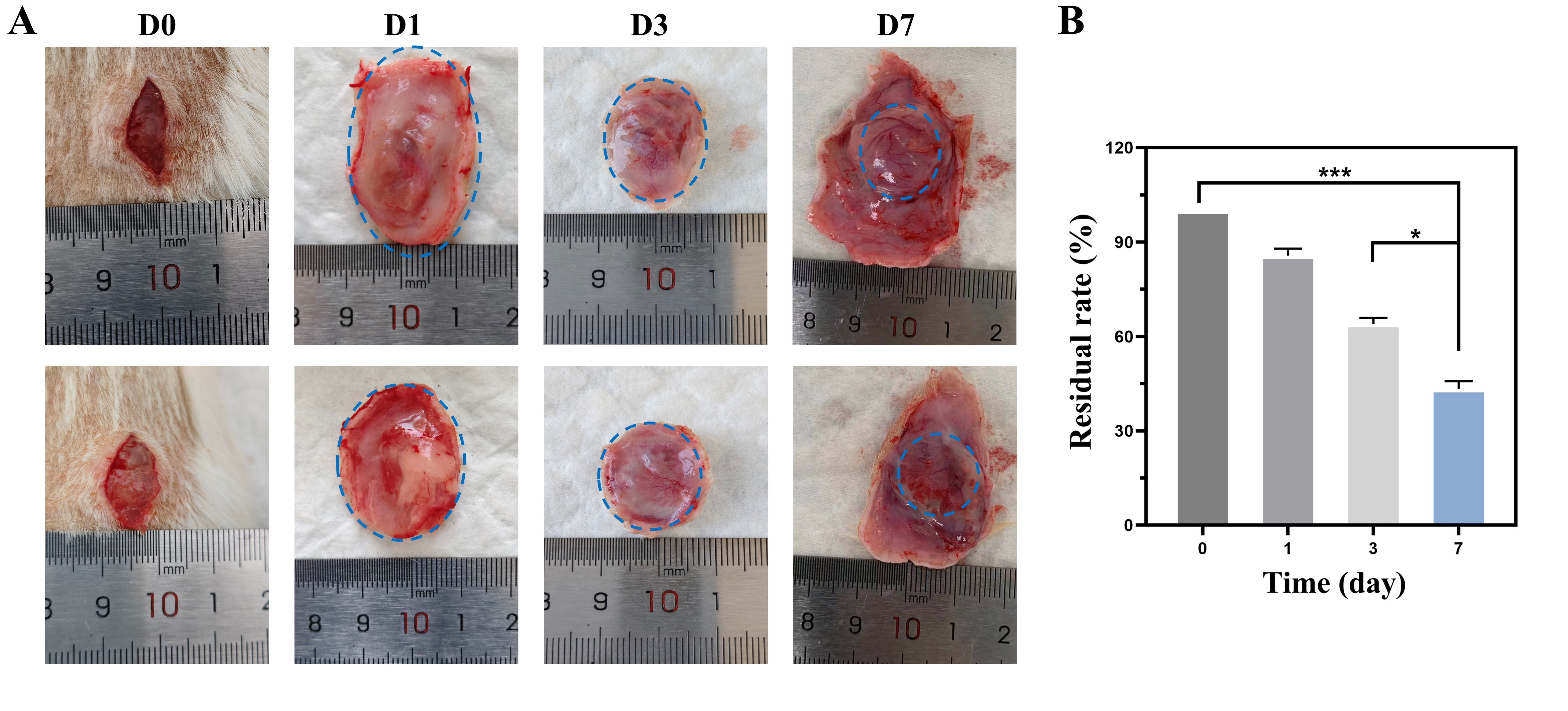


**Fig. S24** The *in vivo* degradation behavior of Cis/Mn/NH@PGP hydrogel in mice after subcutaneous injection for 0, 1, 3 and 7 days. (A) Representative images of hydrogel degradation after subcutaneous injection. (B) Quantitative analysis of residual percentage of hydrogel. *P < 0.05, ***P < 0.001.


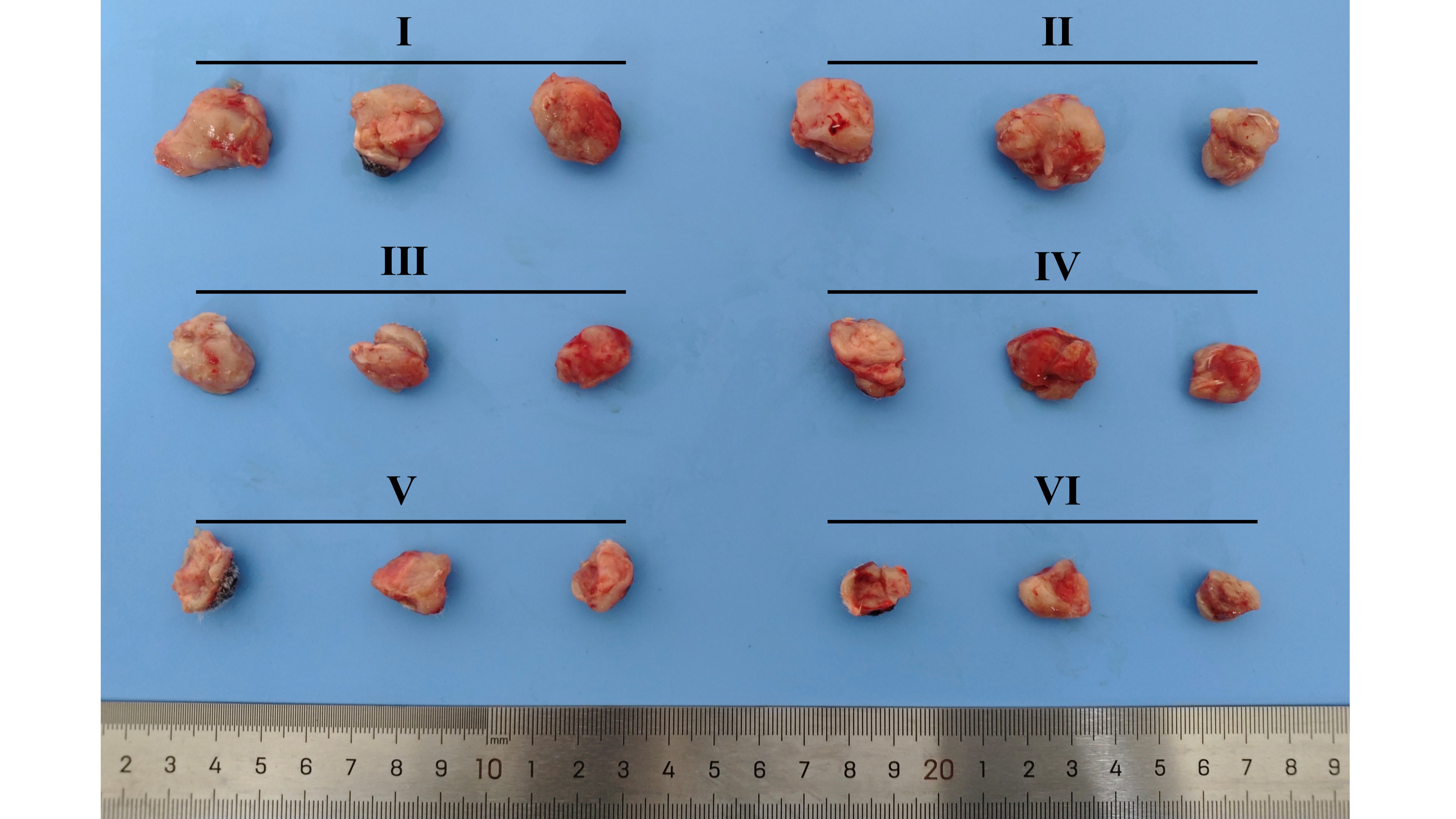


**Fig. S25** Representative images of tumor tissue in K7M2-bearing OS model after treatment with different materials for 24 days. Ⅰ: Control, Ⅱ: PGP, Ⅲ: Mn@PGP, Ⅳ: Cis/Mn/NH@PGP, Ⅴ: Mn/NH@PGP, Ⅵ: Cis/Mn/NH@PGP.


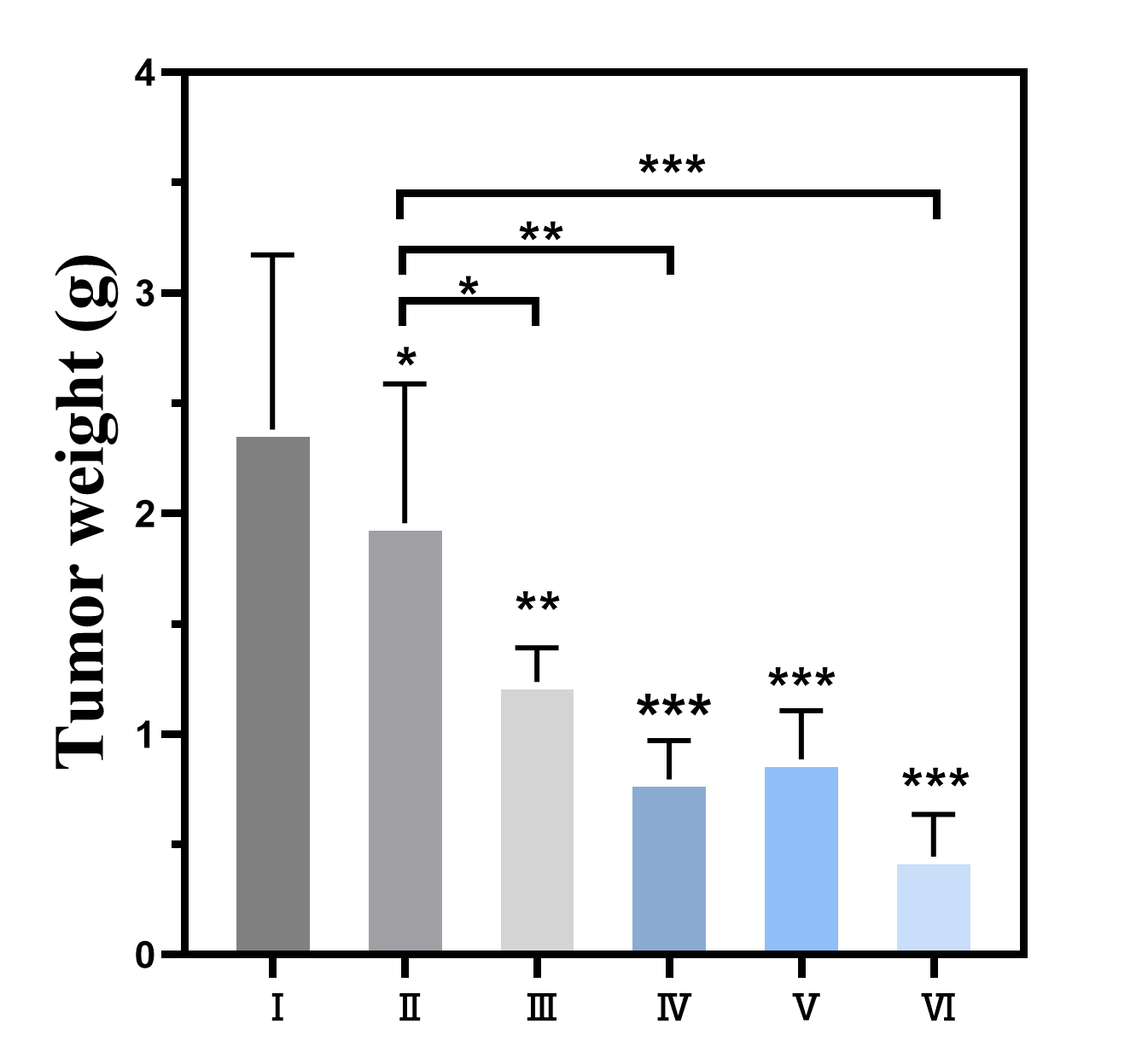


**Fig. S26** The subcutaneous tumor weight in K7M2-bearing OS model after treatment with different materials for 24 days. Ⅰ: Control, Ⅱ: PGP, Ⅲ: Mn@PGP, Ⅳ: Cis/Mn@PGP, Ⅴ: Mn/NH@PGP, Ⅵ: Cis/Mn/NH@PGP. *P < 0.05, **P < 0.01, ***P < 0.001, compared with Control group.


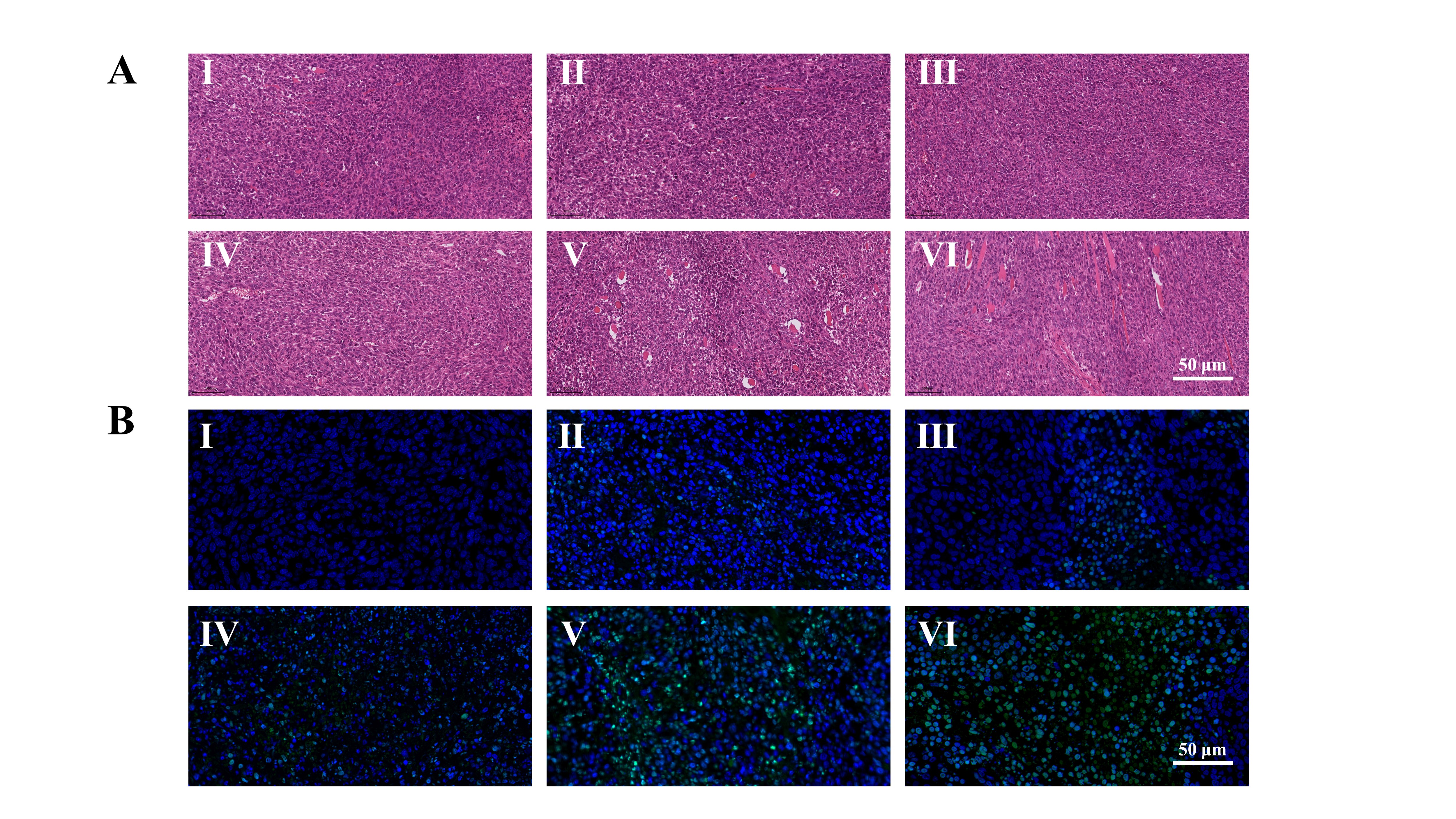


**Fig. S27** (A) H&E staining of subcutaneous tumors in mice after treatment with different materials (B) Immunofluorescence staining of TUNEL. Ⅰ: Control, Ⅱ: PGP, Ⅲ: Mn@PGP, Ⅳ: Cis/Mn@PGP, Ⅴ: Mn/NH@PGP, Ⅵ: Cis/Mn/NH@PGP.


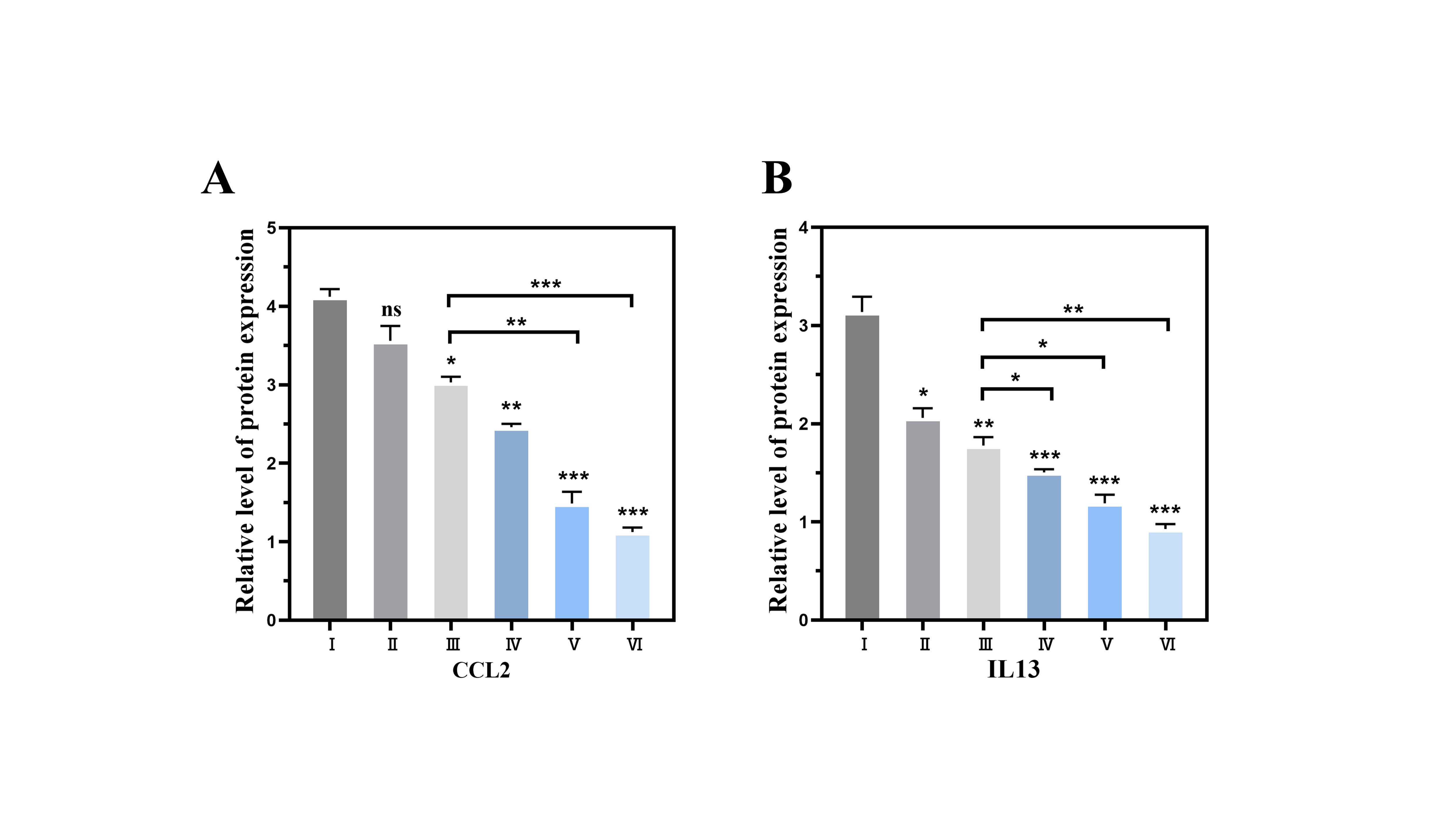


**Fig. S28** The expression levels of CCL2 and IL13 protein in subcutaneous tumor tissue at 24 days. (A) CCL2. (B) IL13. Ⅰ: Control, Ⅱ: PGP, Ⅲ: Mn@PGP, Ⅳ: Cis/Mn@PGP, Ⅴ: Mn/NH@PGP, Ⅵ: Cis/Mn/NH@PGP. ns: no significance, *P < 0.05, **P < 0.01, ***P < 0.001, compared with Control group.


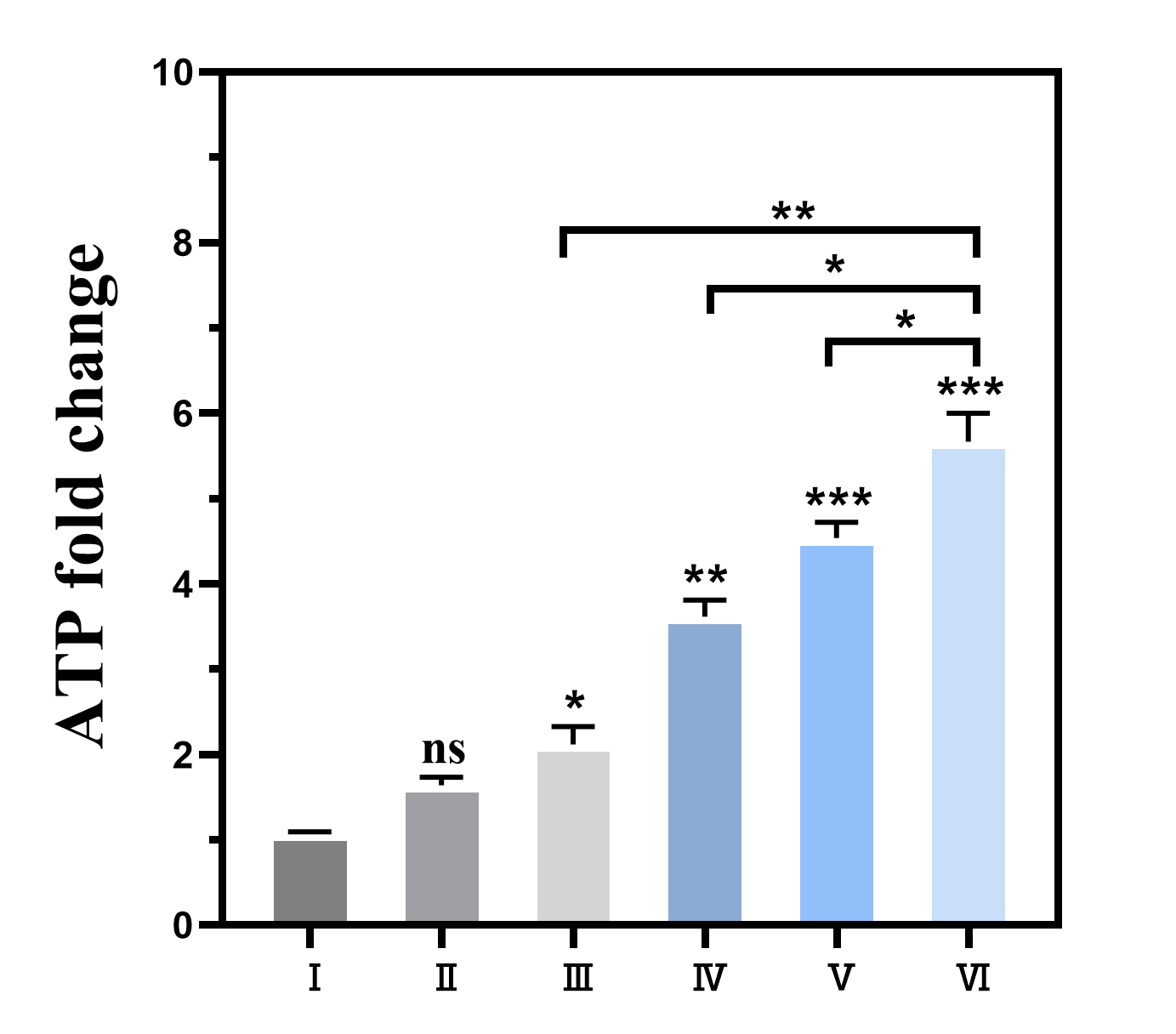


**Fig. S29** The ATP levels in subcutaneous tumor tissue after treatment with different materials for 24 days. Ⅰ: Control, Ⅱ: PGP, Ⅲ: Mn@PGP, Ⅳ: Cis/Mn/NH@PGP, Ⅴ: Mn/NH@PGP, Ⅵ: Cis/Mn/NH@PGP. ns: no significance, *P < 0.05, **P < 0.01, ***P < 0.001, compared with Control group.


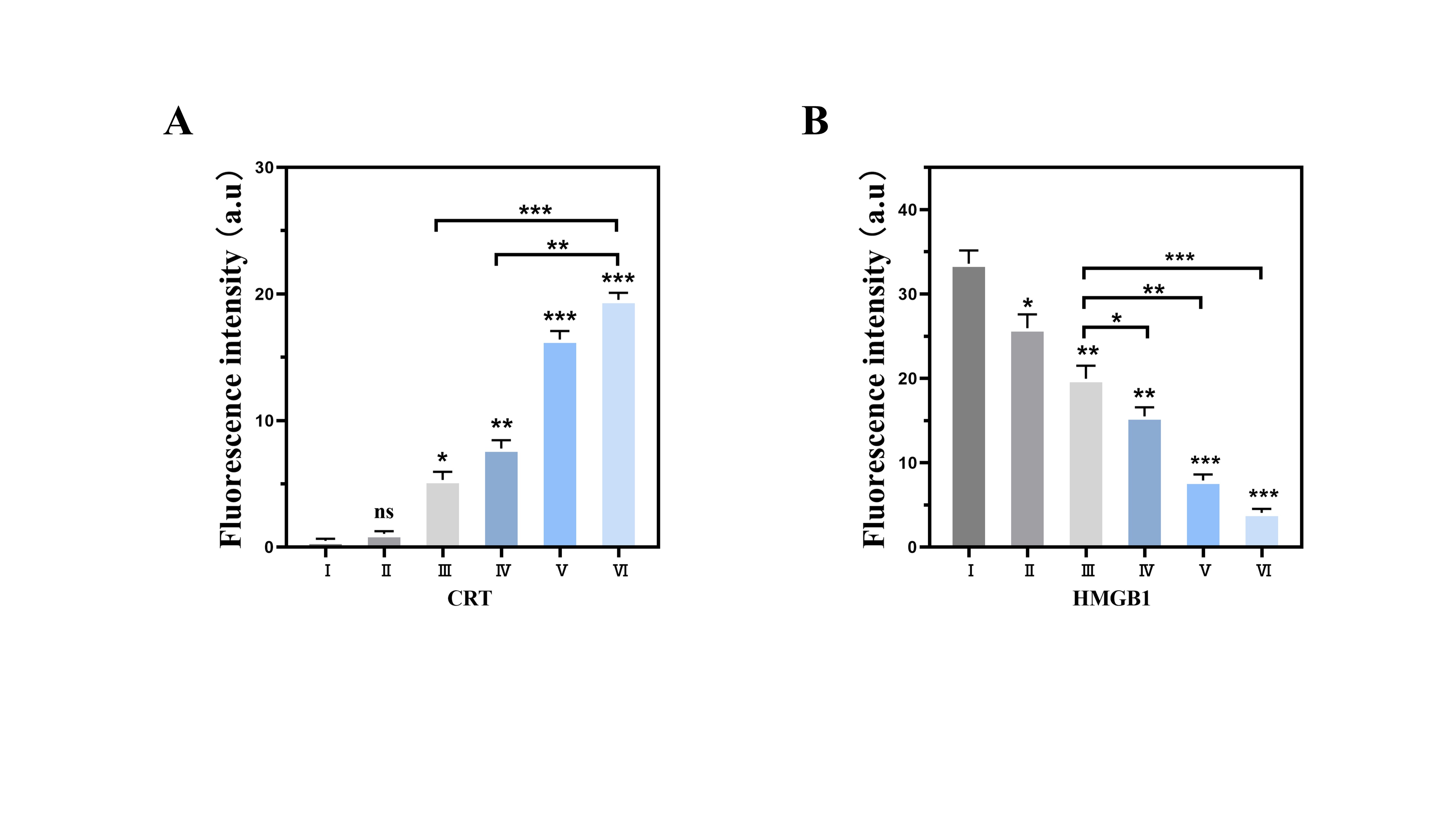


**Fig. S30** Quantification of fluorescence signals of (A) CRT and (B) HMGB1 staining in subcutaneous tumor tissues. Ⅰ: Control, Ⅱ: PGP, Ⅲ: Mn@PGP, Ⅳ: Cis/Mn@PGP, Ⅴ: Mn/NH@PGP, Ⅵ: Cis/Mn/NH@PGP. ns: no significance, *P < 0.05, **P < 0.01, ***P < 0.001, compared with Control group.


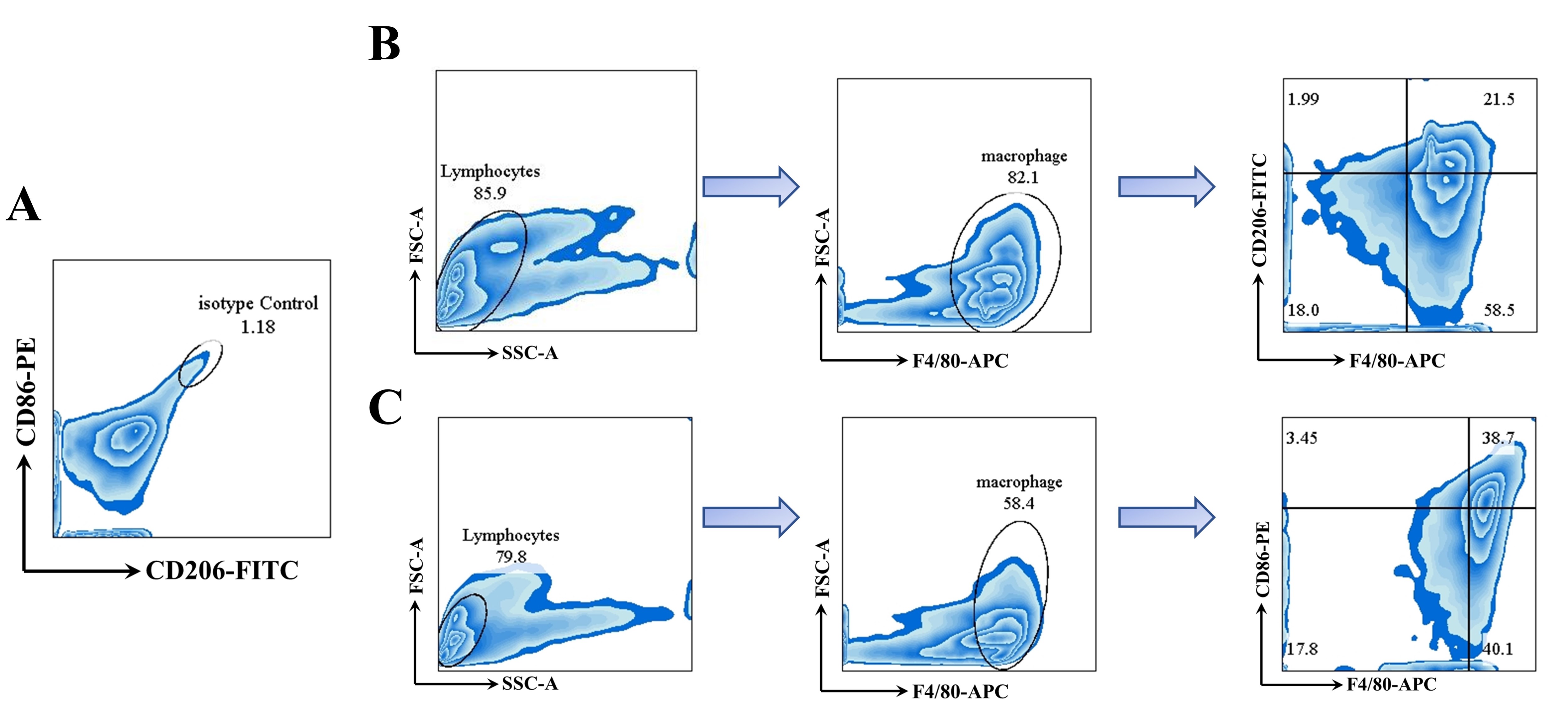


**Fig. S31** (A) Isotype control and (B, C) gating strategy for flow cytometry analysis of M1 marker expression (F4/80⁺/CD86⁺) and M2 marker expression (F4/80⁺/CD206⁺).


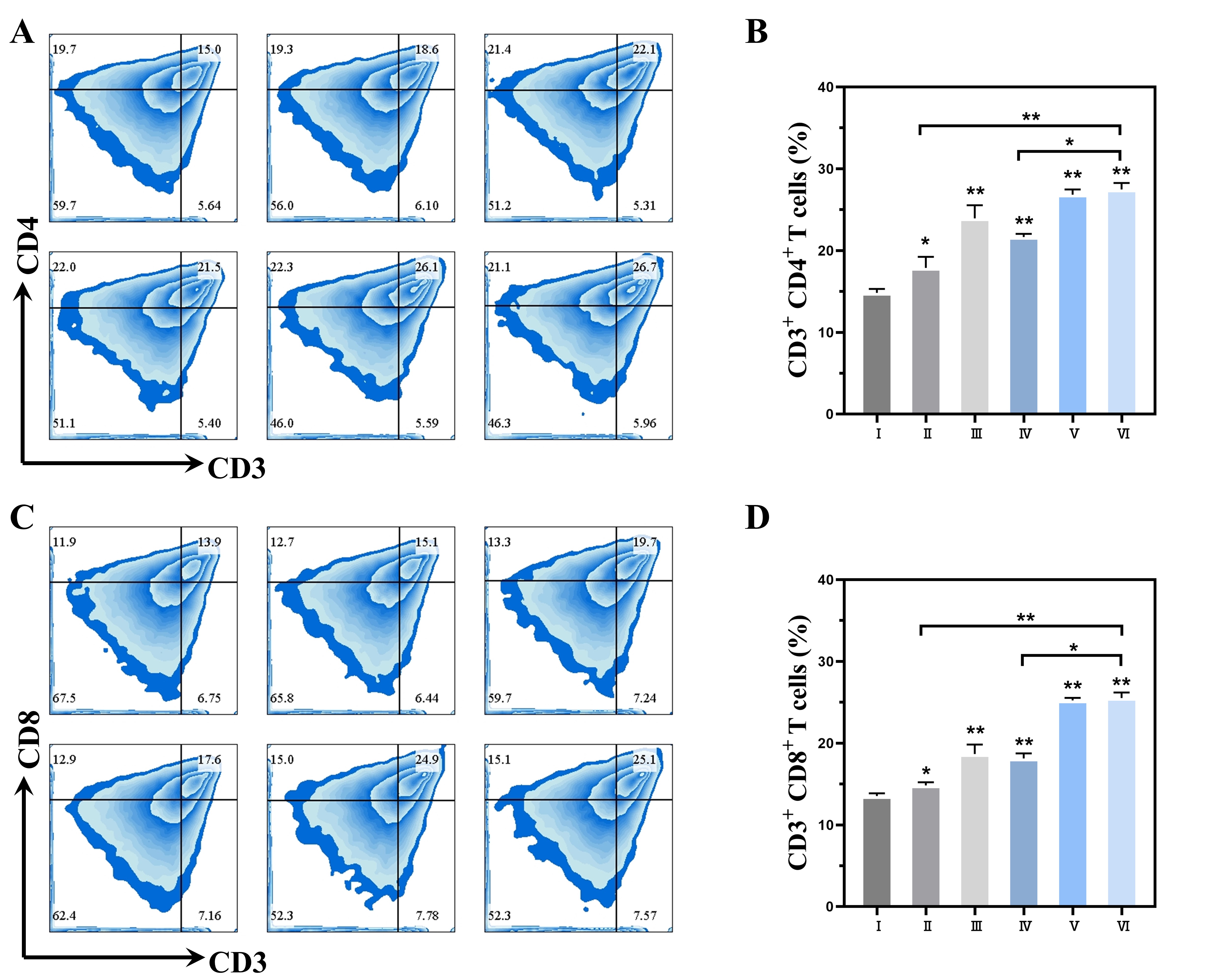


**Fig. S32** Determination of CD3^+^CD4^+^ T cells and CD3^+^CD8^+^ T cells in subcutaneous tumor tissue after treatment with different materials for 24 days by flow cytometry. (A) Flow cytometry plots of CD3^+^CD4^+^ T cells. (B) Quantitative analysis of CD3^+^CD4^+^ T cells. (C) Flow cytometry plots of CD3^+^CD8^+^ T cells. (D) Quantitative analysis of CD3^+^CD8^+^ T cells. Ⅰ: Control, Ⅱ: PGP, Ⅲ: Mn@PGP, Ⅳ: Cis/Mn@PGP, Ⅴ: Mn/NH@PGP, Ⅵ: Cis/Mn/NH@PGP. *P < 0.05, **P < 0.01, compared with Control group.

**S3. Supplementary Table**

**Table S1** Primer sequences of macrophage phenotype-related genes in RAW264.7.

| Gene | Primer sequences |
| --- | --- |
| Arg-1 | Forward: 5'-CAGCAAAGCAGACAGAACTAAG-3' |
|  | Reverse: 5'-AGAAAGGAACTGCTGGGATAC-3' |
| iNOS | Forward: 5'-GAGACAGGGAAGTCTGAAGCAC-3' |
|  | Reverse: 5'-CCAGCAGTAGTTGCTCCTCTTC-3' |
| CD206 | Forward: 5'-TCAATGCCACTGCCATGCCTAC-3' |
|  | Reverse: 5'-AGCTTGCCGTGCGTCTTGC-3' |
| GAPDH | Forward: 5'-CATCACTGCCACCCAGAAGACTG-3' |
|  | Reverse: 5'-ATGCCAGTGAGCTTCCCGTTCAG-3' |

**Supplementary References**

[1] J. Li, F. Han, J. Ma, H. Wang, J. Pan, G. Yang, H. Zhao, Z. Jiayue, J. Liu, Z. Liu, B. Li, Targeting Endogenous Hydrogen Peroxide at Bone Defects Promotes Bone Repair, Adv. Funct. Mater. 32 (2021) 2111208.

[2] T.S. van Solinge, K.P. Friis, K. O'Brien, R.L. Verschoor, J. van Aarle, A. Koekman, X.O. Breakefield, P. Vader, R. Schiffelers, M. Broekman, Heparin interferes with the uptake of liposomes in glioma, Int J Pharm X 6 (2023) 100191.

[3] L. Qi, Q. Luo, Y. Zhang, F. Jia, Y. Zhao, F. Wang, Advances in Toxicological Research of the Anticancer Drug Cisplatin, Chem. Res. Toxicol. 32 (8) (2019) 1469-1486.

[4] Z. Zheng, C. Lei, H. Liu, M. Jiang, Z. Zhou, Y. Zhao, C.Y. Yu, H. Wei, A ROS-Responsive Liposomal Composite Hydrogel Integrating Improved Mitochondrial Function and Pro-Angiogenesis for Efficient Treatment of Myocardial Infarction, Adv. Healthc. Mater. 11 (19) (2022) e2200990.

[5] J. Jiang, R. Wang, L. Yang, Y. Sha, S. Zhao, J. Guo, D. Chen, Z. Zhong, F. Meng, IL-11Rα-targeted nanostrategy empowers chemotherapy of relapsed and patient-derived osteosarcoma, J. Control. Release 350 (2022) 460-470.

[6] C. Zhang, M.H. Hsieh, S.Y. Wu, S.H. Li, J. Wu, S.M. Liu, H.J. Wei, R.D. Weisel, H.W. Sung, R.K. Li, A self-doping conductive polymer hydrogel that can restore electrical impulse propagation at myocardial infarct to prevent cardiac arrhythmia and preserve ventricular function, Biomaterials 231 (2020) 119672.
